# TLR4 abrogates the Th1 immune response through IRF1 and IFN-β to prevent immunopathology during *L*. *infantum* infection

**DOI:** 10.1371/journal.ppat.1008435

**Published:** 2020-03-25

**Authors:** Laís Amorim Sacramento, Luciana Benevides, Sandra Regina Maruyama, Lucas Tavares, Kiyoshi Ferreira Fukutani, Marcela Francozo, Tim Sparwasser, Fernando Queiroz Cunha, Roque Pacheco Almeida, João Santana da Silva, Vanessa Carregaro

**Affiliations:** 1 Department of Biochemistry and Immunology, Ribeirão Preto Medical School, University of São Paulo, Ribeirão Preto; 2 Fiocruz-Bi-Institutional Translational Medicine Project, Brazil; 3 Center for Biological and Health Sciences, Federal University of São Carlos, São Carlos, Brazil; 4 Department of Cellular and Molecular Biology, Ribeirão Preto Medical School, University of São Paulo, Ribeirão Preto, Brazil; 5 Institute of Infection Immunology, Centre for Experimental and Clinical Infection Research (Twincore), Hannover Medical School (MHH) and Helmholtz Centre for Infection Research (HZI), Hannover, Germany; 6 Department of Medical Microbiology and Hygiene, University Medical Center of the Johannes Gutenberg University Mainz, Mainz, Germany; 7 Department of Pharmacology, Ribeirão Preto Medical School, University of São Paulo, Ribeirão Preto, Brazil; 8 Center for Biology and Health Sciences, Federal University of Sergipe, Aracaju, SE, Brazil; Queensland Institute of Medical Research, AUSTRALIA

## Abstract

A striking feature of human visceral leishmaniasis (VL) is chronic inflammation in the spleen and liver, and VL patients present increased production levels of multiple inflammatory mediators, which contribute to tissue damage and disease severity. Here, we combined an experimental model with the transcriptional profile of human VL to demonstrate that the TLR4-IFN-β pathway regulates the chronic inflammatory process and is associated with the asymptomatic form of the disease. *Tlr4*-deficient mice harbored fewer parasites in their spleen and liver than wild-type mice. TLR4 deficiency enhanced the Th1 immune response against the parasite, which was correlated with an increased activation of dendritic cells (DCs). Gene expression analyses demonstrated that IRF1 and IFN-β were expressed downstream of TLR4 after infection. Accordingly, IRF1- and IFNAR-deficient mice harbored fewer parasites in the target organs than wild-type mice due to having an increased Th1 immune response. However, the absence of TLR4 or IFNAR increased the serum transaminase levels in infected mice, indicating the presence of liver damage in these animals. In addition, IFN-β limits IFN-γ production by acting directly on Th1 cells. Using RNA sequencing analysis of human samples, we demonstrated that the transcriptional signature for the TLR4 and type I IFN (IFN-I) pathways was positively modulated in asymptomatic subjects compared with VL patients and thus provide direct evidence demonstrating that the TLR4-IFN-I pathway is related to the nondevelopment of the disease. In conclusion, our results demonstrate that the TLR4-IRF1 pathway culminates in IFN-β production as a mechanism for dampening the chronic inflammatory process and preventing immunopathology development.

## Author summary

Visceral leishmaniasis (VL) is one of the most lethal neglected tropical diseases and is caused by *Leishmania* parasites. Most subjects infected with *Leishmania* present subclinical VL symptoms, and their immune response is mediated by Th1 cells and immunoregulatory mechanisms. However, when infection progresses to disease, VL patients present increased levels of inflammatory mediators in the serum which are related to the severity of disease. During infection, Toll-like receptors (TLRs) interact with *Leishmania* parasites and contribute to the outcome of the disease. Herein, we report that TLR4 signaling hampers the chronic immune response during VL to prevent immunopathology. TLR4 triggers the activation of IRF1 and thus induces the transcription of IFN-β, which in turn acts directly on Th1 cells to limit the production of IFN-γ. In addition, a transcription analysis of human VL samples provides direct evidence demonstrating that the TLR4-IFN-I pathway is related to the asymptomatic form of the disease. Collectively, our findings reveal that TLR4 hampers the Th1 immune response through IRF1 and IFN-β to prevent immunopathology during VL.

## Introduction

Leishmaniasis is a broad-spectrum disease caused by the intracellular protozoan parasite *Leishmania spp*. Among the clinical manifestations of this disease, visceral leishmaniasis (VL) is the most severe form and causes high human mortality and morbidity around the world. VL, which results from infection with *Leishmania donovani* and *Leishmania infantum*, affects mainly the visceral organs and induces a chronic and potentially fatal pathology if left untreated following clinical diagnosis.

*Leishmania* species have evolved a wide variety of strategies to evade host immune mechanisms and thus maintain a chronic infectious state and survive within the host [1,2]. Approximately 90% of subjects infected with *Leishmania* present subclinical VL symptoms and are considered asymptomatic because they exhibit a balance between effector mechanisms related to the development of a Th1-mediated immune response against *Leishmania* antigens and immunoregulatory mechanisms [3]. Once the infection progresses to disease, patients present a predominance of anti-inflammatory mediators, such as IL-10, which can encourage parasite multiplication and interfere with infection control. This condition contributes to enlargement of the spleen and liver and might cause hematological disorders [4]. In addition, VL patients exhibit increased production levels of multiple pro- and anti-inflammatory cytokines and chemokines, which are closely associated with tissue damage and disease severity [5–8]. This finding indicates that immune evasion or other immunosuppressive mechanisms contribute to the pathogenesis of infection.

Host-parasite interactions during the innate immune response determine the fate of adaptive immunity and contribute to parasite persistence or replication control in leishmaniasis [9]. In this context, dendritic cells (DCs) are the major innate cells that respond to *Leishmania* infection, and these cells trigger an effective adaptive response due to their wide repertoire of pattern recognition receptors (PRRs) [10,11]. Otherwise, *Leishmania* infection can hamper DC function through the manipulation of different pathways, such as inhibition of their migration to T cell-containing areas of the spleen due to a loss of CCR7 expression [12], inhibition of cross-presentation [13], inhibition of IL-12 production by HIF-1α [14] and IL-10 production by T cells [15]. *L*. *infantum* parasites infect and survive inside DCs [16] and inhibit DC apoptotic death, and this mechanism enables their spread to organs such as the bone marrow and liver [17].

Toll-like receptors (TLRs) are thought to be among the most ancient pathogen recognition systems [18], and these receptors directly interact with *Leishmania* species during the initial sensing of these parasites and mount the subsequent adaptive immune response [19]. Our group previously demonstrated that TLR2 and TLR9 act cooperatively during induction of the protective immune response, specifically during the development of Th1 and Th17 immune responses. Both receptors contribute to the activation of DCs and the secretion of important mediators for infection control [20,21].

TLR4 plays a role in the control of *Leishmania major* infection [22,23] and is able to detect glycosphingophospholipid (GSPL) from *L*. *donovani* and proteoglycolipid complex (P8GLC) from *Leishmania pifanoi* [24,25]. In the context of VL, *Tlr4*^-/-^ mice infected with *L*. *donovani* exhibit deficient production of proinflammatory cytokines, which culminates in increased parasite replication in the target organs of the disease [26]. Moreover, *L*. *donovani* has devised mechanisms to alter the TLR4 signaling pathway to favor establishment of infection [27,28]. The role of TLR4 during VL caused by *L*. *infantum* remains to be elucidated. We herein demonstrate that the TLR4 signaling pathway abrogates the Th1 immune response through an interferon regulatory factor 1 (IRF1)- and interferon alpha/beta receptor (IFNAR)-dependent mechanism to avoid tissue damage. In human samples, the gene signature for TLR4 and type I IFN (IFN-I) was found to be positively modulated in asymptomatic subjects compared with VL patients. Taken together, our results reveal the relevance of the TLR4-IRF1 pathway culminating in IFN-β production as a mechanism for attenuating the immune response and consequently preventing immunopathology development.

## Results

### TLR4 deficiency strengthens the Th1 response and thereby interferes with parasite control during experimental VL

**Fig 1:**
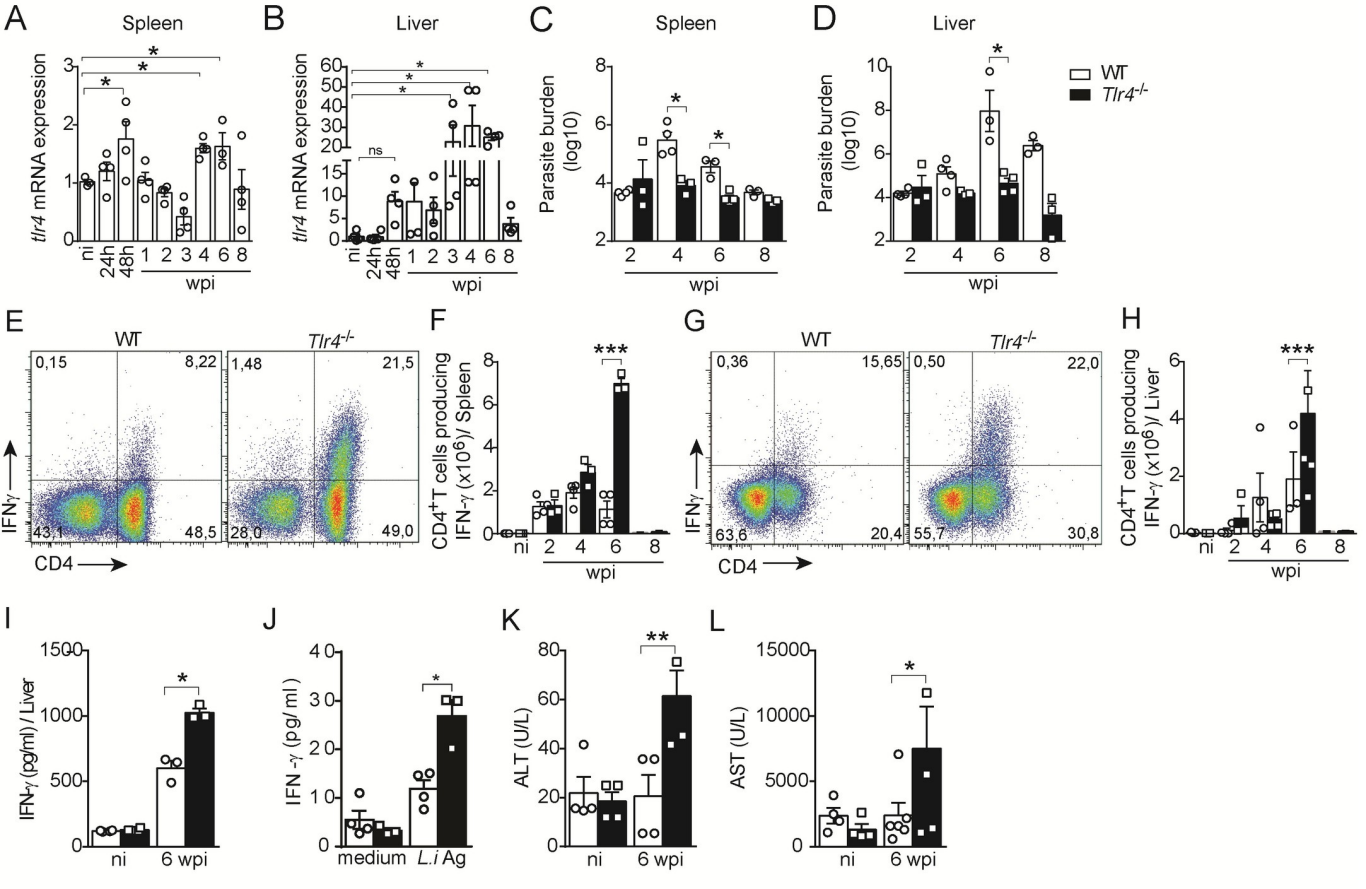
TLR4 deficiency strengthens the Th1 response and restrains parasites in target organs. (A and B) mRNA expression of *tlr4* in the spleen and liver of naïve and *L*. *infantum*-infected C57BL/6 WT mice at the indicated time points after infection. (C-L) C57BL/6 WT and *Tlr4*^*-/-*^ mice were infected with 10^7^ *L*. *infantum* promastigotes. (C and D) Parasite loads in the spleen and liver at 2, 4, 6 and 8 wpi. (E and G) Representative dot plots of IFN-γ production by CD4^+^ T cells from the spleen (E) and liver (G) of *L*. *infantum*-infected WT and TLR4^-/-^ mice in response to polyclonal restimulation at 6 wpi. (F and H) The graph bars represent the absolute numbers of IFN-γ-producing CD4^+^ T cells in the spleen (F) and liver (H) of naïve (ni) and *L*. *infantum*-infected WT and TLR4^-/-^ mice at the indicated weeks postinfection. (I) IFN-γ levels in the liver homogenate supernatant at 6 wpi. (J) IFN-γ levels in the supernatants from splenocytes restimulated with *L*. *infantum* antigen or medium for 72 h at 6 wpi. (K and L) ALT and AST levels in the sera of naïve and *L*. *infantum*-infected WT and *Tlr4*^-/-^ mice at 6 wpi. The data are expressed as the means ± SEMs and are representative of three independent experiments (n = 3–4 mice). The statistical significance was calculated by one-way ANOVA with the Bonferroni post hoc test (*p < 0.05, **p < 0.01, and ***p < 0.001).

The kinetics of *Tlr4* expression in the target organs of *L*. *infantum*-infected wild-type (WT) C57BL/6 mice were evaluated. High levels of *Tlr4* messenger RNA (mRNA) expression were detected initially after 48 h of infection and later at 4 and 6 weeks post-infection (wpi), in the spleen (Fig 1A). In the liver, *tlr4* expression largely occurred in only the chronic stage of infection, starting at 3 wpi and remaining high up to 6 wpi (Fig 1B). We monitored the expression of TLR4 on several leucocytes, including macrophages, DCs, and T and B cells from the spleen of WT mice at 48 h and 6 wpi, which are periods in which mRNA TLR4 expression was detected in the spleen of infected WT mice. Among all the cells analyzed, the highest expression of TLR4 was observed on DCs (S2 Fig).

To characterize the role of TLR4, *Tlr4*^-/-^ mice and littermate controls were infected with *L*. *infantum* parasites, and the parasitic loads in the target organs were quantified at different time points postinfection. Interestingly, compared with the littermate controls, the *Tlr4*^-/-^ mice harbored significantly lower parasite loads in the spleen at 4 and 6 wpi (Fig 1C), whereas the liver parasite load was reduced at 6 wpi (Fig 1D). The control of parasite growth was strictly associated with higher numbers of CD4^+^ T cells producing IFN-γ at 6 wpi in the spleen (Fig 1E and 1F) and liver (Fig 1G and 1H). In addition, the liver from the infected *Tlr4*^-/-^ mice contained significantly higher concentrations of IFN-γ than that from the infected WT mice (Fig 1I). The effect of TLR4 on IFN-γ production was parasite specific because the TLR4-deficient group exhibited a significantly higher level of IFN-γ in the supernatant of splenocytes cultured *in vitro* with crude *L*. *infantum* antigen compared with that in the WT group (Fig 1J). Nevertheless, heightened levels of alanine transaminase (ALT) and aspartate transaminase (AST) were detected in the infected *Tlr4*^−/−^ mice (Fig 1K and 1L, respectively), which indicated the presence of liver damage in these animals. Altogether, these results are consistent with the hypothesis that TLR4 promotes parasite persistence by affecting the Th1 pattern of the immune response and preventing pathological tissue damage.

### The DC activation profile during *L*. *infantum* infection is enhanced in TLR4-deficient mice

**Fig 2:**
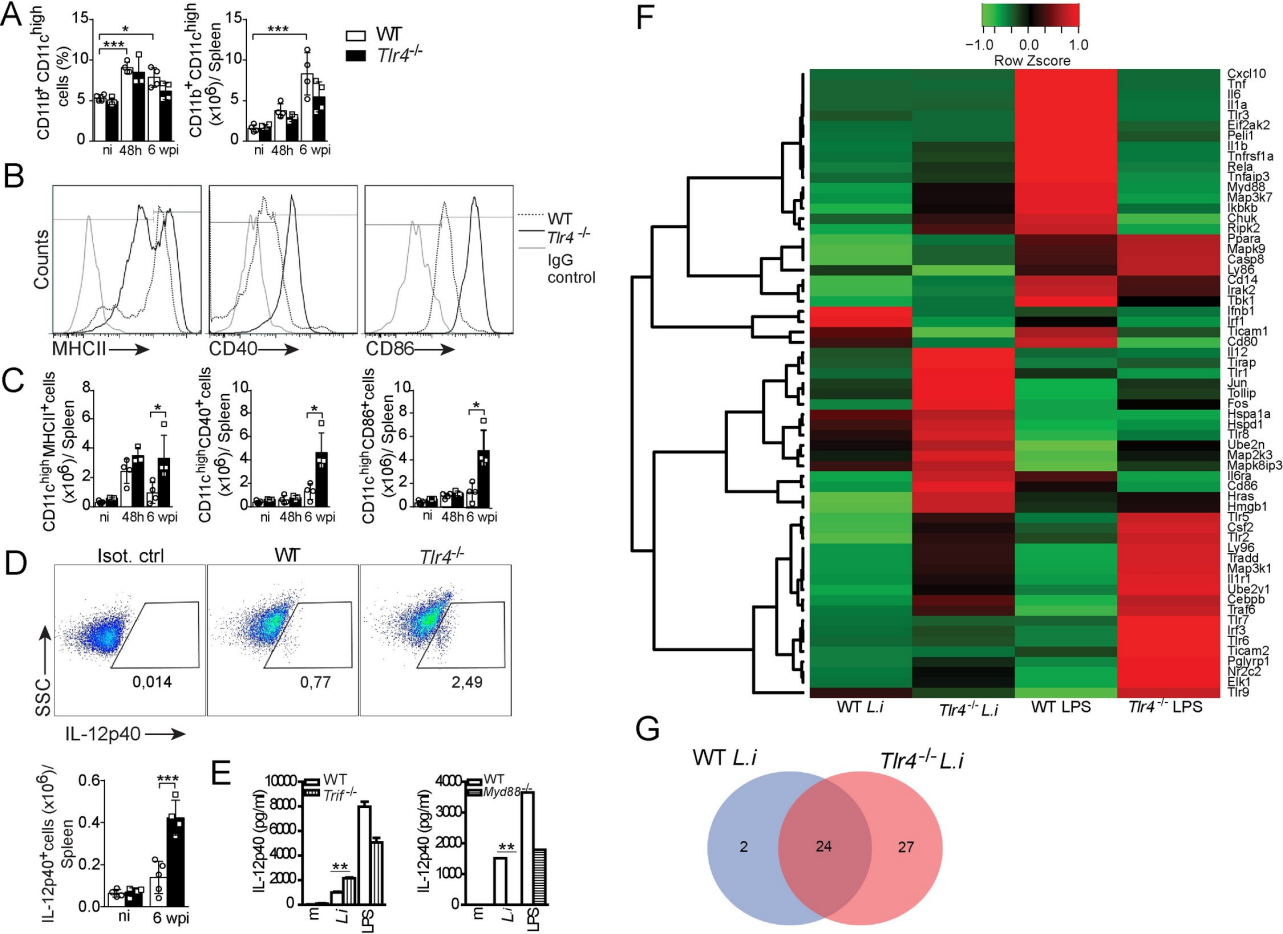
CD11b^+^ CD11c^high^ cell activation during *L*. *infantum* infection is enhanced in TLR4-deficient mice. (A-C) WT and *Tlr4*^-/-^ mice were i.v. infected with 10^7^ *L*. *infantum* parasites and euthanized at 48 h or 6 wpi. (A) Percentage and absolute number of CD11b^+^ CD11c^high^ cells. (B) Representative histograms of MHCII, CD40 and CD86 expression in CD11b^+^CD11c^high^ cells from the spleens of *L*. *infantum*-infected mice. (C) Absolute numbers of MHCII-, CD40- and CD86-expressing splenic CD11b^+^ CD11c^high^ cells. (D) Representative dot plot and absolute number of CD11b^+^ CD11c^high^ cells expressing IL-12p40 at 6 wpi. (E) Bone marrow-derived dendritic cells (BMDCs) from WT, *Trif*^-/-^ and *Myd88*^-/-^ mice stimulated for 24 h with *L*. *infantum* (5 parasites:1 cell) or LPS (200 ng/mL). (F and G) Gene expression profiles of WT and *Tlr4*^-/-^ BMDCs stimulated with *L*. *infantum*, LPS or medium alone for 4 h as determined by a PCR array. (F) Heat map representing the relative fold changes in the expression levels of TLR-induced genes normalized to that of β-actin; the genes are clustered by distance (hclust, gplot from R package). (G) Venn diagram of transcripts showing a 2-fold increase or decrease in expression. The data are expressed as the means ± SEMs (n = 4 mice in A-D; BMDCs assessed in quadruplicate in E) and are representative of three independent experiments. The PCR array is representative of the quadruplicate sample pool. Statistical significance was calculated by one-way ANOVA with the Bonferroni post hoc test (*p < 0.05, **p < 0.01, and ***p < 0.001).

Given the increased Th1 response observed in infected *Tlr4*^-/-^ mice and the biphasic induction of *Tlr4* mRNA in the spleen at 48 h and 6 wpi, we characterized both the number and the activation profile of DCs at different stages of infection: early (i.e., 48 h postinfection) and late (6 wpi). The percentage and total number of spleen CD11b^+^CD11c^high^ cells at 6 weeks after *L*. *infantum* infection were increased in both the WT and *Tlr4*^-/-^ mice compared with the respective naïve group (Fig 2A). The analysis of DC activation revealed that the MHC-II levels on the surface of CD11b^+^ CD11c^high^ cells were increased at 48 h postinfection, and similar findings were obtained in the TLR4^-/-^ mice and their littermate controls. The most significant difference in the total CD11b^+^ CD11c^high^ cells expressing MHCII, CD40 and CD86 was detected at 6 wpi in the TLR4^-/-^ mice (Fig 2B and 2C). In addition, *Tlr4*^-/-^ mice presented increased production of IL-12p40 by spleen CD11b^+^CD11c^high^ cells compared with the WT mice (Fig 2D). This finding indicates that the absence of TLR4 enhances the DC activation profile at the chronic stage of infection.

Because TLR4 can signal through the MyD88 or TRIF signaling pathways [29], we investigated which adaptor molecule might be involved in TLR4-mediated immunomodulatory activity in *in vitro* bone marrow-derived dendritic cells (BMDCs) infected with parasite. In these cells, IL-12p40 has been determined to be an essential cytokine for overcoming *L*. *infantum* infection through Th1 subset differentiation [30]. Lipopolysaccharide (LPS) and medium were used as the positive and negative controls, respectively. As expected, the parasite-infected WT BMDCs released significantly higher levels of IL-12p40 than the medium-treated WT BMDCs. Interestingly, the absence of TRIF enhanced the ability of infected DCs to produce IL-12p40 (Fig 2E). We did not further explore the role of MYD88 in the immunomodulatory function of TLR4 because the production of IL-12 in response to *L*. *infantum* was blunted in the absence of MYD88 (Fig 2E). Additionally, the IL-12p40 levels after stimulation with LPS were significantly decreased in both the *Trif*^*-/-*^ and *Myd88*^*-/-*^ BMDCs compared with the WT BMDCs (Fig 2E), which demonstrates the specificity of TLR4 during parasite infection.

Because TLR4 acts as a coreceptor for CD14 in addition to MyD88 and TRIF [31], we verified the role of CD14 during *L*. *infantum* infection and found no difference in the expression of MHCII and CD86 by splenic DCs between the WT and *Cd14*^*-/-*^ mice (S3A–S3C Fig). In addition, no differences in IFN-γ production by CD4^+^ T cells (S3D–S3F Fig) or in the parasite burden in the target organs were found between these mice (S3H Fig), which demonstrates that the immunomodulatory function of TLR4 during *L infantum* infection is independent of CD14 (S3 Fig). Collectively, our results suggest that TLR4 downregulates DC activation through the TRIF signaling pathway and that this effect could affect the development of the Th1 response and favor parasite persistence.

### *L*. *infantum* parasites promote an alteration in the transcriptional signature of dendritic cells in a TLR4-dependent manner

To obtain more evidence demonstrating the role of TLR4 in the immunomodulatory function of DCs during *L*. *infantum* infection, we obtained and analyzed the transcriptional patterns of genes related to TLR signaling in both WT and *Tlr4*^-/-^ BMDCs infected with *L*. *infantum*. LPS-stimulated BMDCs were used as a positive control. Gene expression analyses were performed by PCR array, and the experimental expression was compared with that of unstimulated WT BMDCs.

A total of 84 genes were screened, and *L*. *infantum* infection induced a slight upregulation of the transcripts in the WT BMDCs. mRNAs for *Ifnb1*, *Irf1*, *Ticam1*, *Hspa1a*, *Mapk8ip3*, and *Hmgb1* were among the most upregulated transcripts. However, the absence of TLR4 altered the gene profile, upregulating mRNAs for *Il12*, *Tirap*, *Tlr1*, *Jun*, *Tollip*, *Fos*, *Map2k3*, *Mapk8ip3*, *Tlr8*, *Il6ra*, *Cd86* and *Hras* (Fig 2F), which are all genes related to DC activation [32–37], demonstrating that the presence of TLR4 reduces DC activation.

Among the significantly modulated transcripts, 27 transcripts were significantly expressed with a ± 2-fold change variation exclusively by the *Tlr4*^-/-^ BMDCs, 24 were shared between the WT and *Tlr4*^-/-^ BMDCs, and 2 were exclusively upregulated by the WT BMDCs (Fig 2G). Among the transcripts exclusively expressed in the WT BMDCs, the mRNA for *Irf1* stands out since it is related to negative regulation of the immune response [38,39] following TLR activation [40].

**Fig 3:**
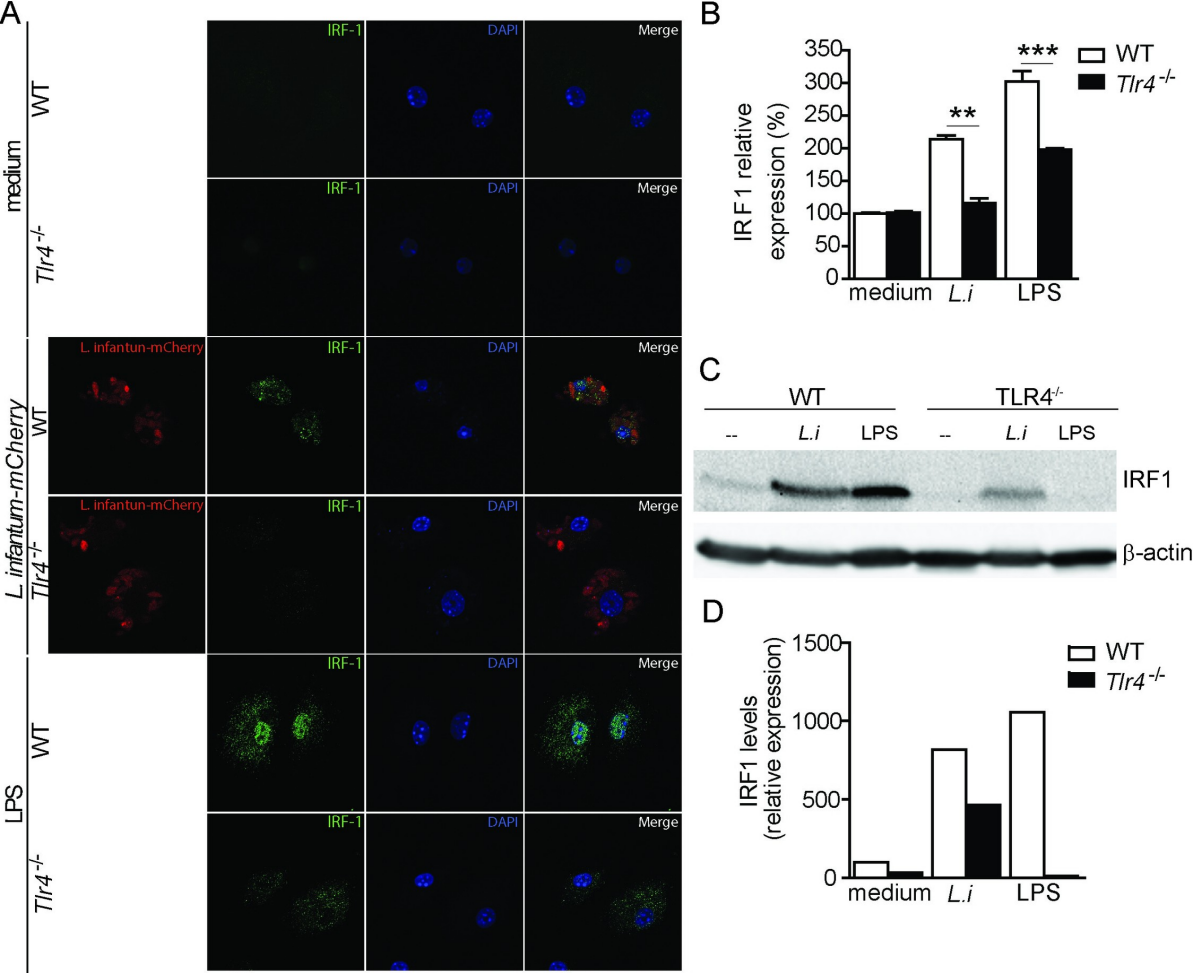
IRF1 expression is dependent on TLR4 during *L*. *infantum in vitro* infection. (A and B) WT and *Tlr4*^-/-^ BMDCs were stimulated with m-Cherry-*L*. *infantum*, LPS or medium alone. (A) BMDCs were stimulated for 24 h, fixed and subjected to immunofluorescent staining for IRF1. (B) IRF1 expression in the experimental group was quantified relative to that in the non-stimulated (medium only) WT group. (C) The cells were lysed after 8 h of stimulation, and the total protein lysates were immunoblotted for IRF1 and β-actin. (D) IRF1 expression in the experimental group was quantified relative to that in the non-stimulated (medium only) WT group. The data are expressed as the means ± SEMs (in quadruplicate) and are representative of two independent experiments. The statistical significance was calculated by one-way ANOVA with the Bonferroni post hoc test (**p < 0.01 and ***p < 0.001).

Confirming the *Irf1* transcription data, *L*. *infantum-mCherry* infection induced IRF1 expression in the WT BMDCs, whereas the *Tlr4*^-/-^ BMDCs displayed significant reduction in protein expression (average of 50% reduction) (Fig 3A and 3B). Reinforcing the immunofluorescence data, IRF1 expression in BMDCs was induced during *L*. *infantum* infection in a TLR4-dependent manner because a significant decrease in IRF1 expression was observed in the parasite-infected *Tlr4*^-/-^ BMDCs compared with the WT BMDCs, as shown by western blot analysis. A similar response was observed in the LPS-stimulated BMDCs, and IRF1 was not expressed in the unstimulated BMDCs (Fig 3C and 3D).

Given the increased expression of costimulatory molecules in *Tlr4*^*-/-*^ splenic DCs, we evaluated the statuses of the coinhibitory molecules PD-L1 and CTLA-4 in these cells. Both the percentages and median fluorescence intensities (MFIs) of these molecules in splenic DCs from the infected WT mice were similar to those in splenic DCs from the infected *Tlr4*^*-/-*^ mice (S4 Fig); thus, we excluded the participation of coinhibitory molecules, such as PD-L1 and CTLA-4, in the control of the Th1-inflammatory process mediated by TLR4.

The TLR4 pathway can induce the phosphorylation, dimerization and nuclear translocation of IRF3 through the recruitment of TRAF3 [40]. In contrast, *Leishmania* infection alters the ubiquitination pattern of TRAF3 required for effective signaling as an evasion mechanism [28]. To investigate the possible participation of IRF3 during *L*. *infantum* infection, BMDCs from WT and *Irf3*^*-/-*^ mice were infected with *L*. *infantum*, and their activation profiles were evaluated. Infected *Irf3*^*-/-*^ BMDCs presented a lower MFI for MHCII (S5A Fig) compared with the infected WT BMDCs, and no difference in the expression of CD86 was observed (S5B Fig). The infected *Irf3*^*-/-*^ BMDCs presented reduced percentages of CD11c^+^ IL-12p40^+^ cells compared with the WT BMDCs (S5C and S5D Fig). Confirming these data, the production of IL-12p40 in the supernatant of infected *Irf3*^*-/-*^ BMDCs was significantly reduced compared with that obtained with infected WT BMDCs (S5E Fig). Thus, we conclude that IRF3 contributes to the activation of DCs and IL-12p40 production and exclude the possibility of its participation in the TLR4 signaling pathway. Taken together, these data demonstrate that IRF1 expression is regulated in a TLR4-dependent manner.

### IRF-1 participates in TLR4 immunomodulatory functions during *in vivo* VL

**Fig 4:**
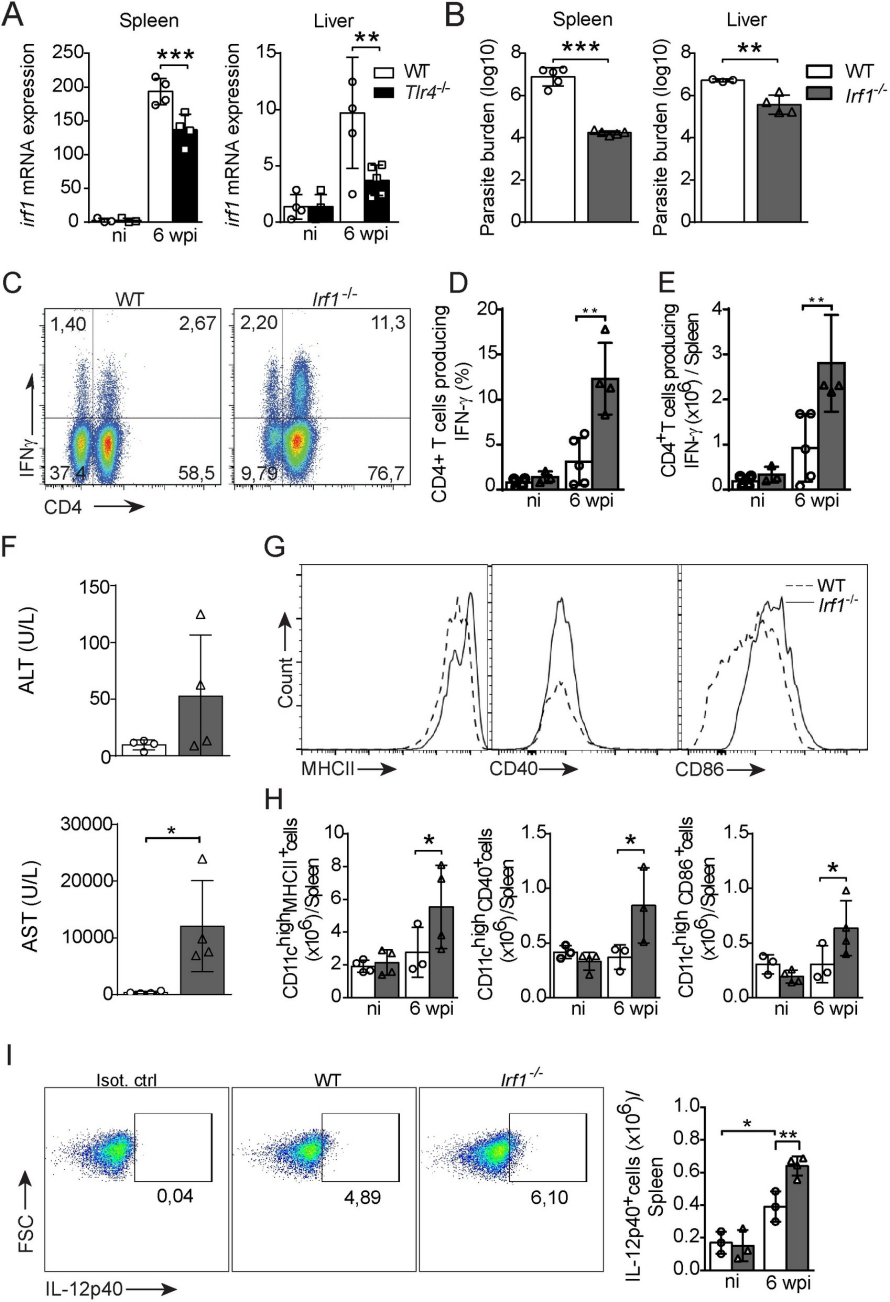
IRF1-deficient mice exhibit a stronger immune response and increased resistance to *L*. *infantum* infection. (A) *Irf1* mRNA expression in the spleens and livers of naïve and *L*. *infantum*-infected WT and *Tlr4*^-/-^ mice. (B-I) WT and *Irf1*^-/-^ mice were i.v. infected with 10^7^ *L*. *infantum* parasites and euthanized at 6 wpi. (B) Parasitic loads in the spleen and liver. (C) Representative dot plots showing the production of IFN-γ by CD4^+^ T cells from the spleens of *L*. *infantum*-infected WT and *Irf1*^-/-^ mice in response to polyclonal restimulation. (D-E) Bar graphs showing the percentages (D) and absolute numbers (E) of IFN-γ-producing CD4^+^ T cells in the spleens of naïve (ni) and *L*. *infantum*-infected *Irf1*^-/-^ and WT mice. (F) ALT and AST levels in the sera of *L*. *infantum*-infected WT and *Irf1*^-/-^ mice at 6 wpi. (G) Representative histograms of CD11b^+^CD11c^high^ cells from the spleen stained for MHCII, CD40 and CD86. (H) Absolute numbers of MHCII-, CD40- and CD86-expressing CD11b^+^ CD11c^high^ cells from naïve and *L*. *infantum*-infected mice. (I) Representative dot plot and bar graph showing the absolute number of CD11b^+^CD11c^high^ cells expressing IL-12p40. The data are expressed as the means ± SEMs (n = 3–5 mice, A-H) and are representative of three independent experiments. Statistical significance was calculated by one-way ANOVA with the Bonferroni post hoc test (A, D, E, G and H) or Student’s t test (B) (*p < 0.05, **p < 0.01, and ***p < 0.001).

Based on our collective observations, we next evaluated whether the expression of IRF1 was TLR4 dependent in the *in vivo* VL model. Indeed, *L*. *infantum* infection induced *Irf1* transcription in both target organs from the WT and *Tlr4*^-/-^ mice, but a significant reduction in the *Irf1* transcript level was observed in the infected *Tlr4*^-/-^ mice compared to the infected WT mice (Fig 4A). Subsequently, we assessed whether IRF1 influences the negative modulation of the Th1 immune response during infection. To this end, the parasite numbers and Th1 response of infected 129/SvEv WT and *Irf1*^-/-^ mice were evaluated at 6 wpi. Similar to those of the infected *Tlr4*^-/-^ mice (Fig 1C), and in contrast to those of their WT counterparts, the spleen and liver of the mice lacking *Irf1* harbored reduced parasite loads (Fig 4B). In addition, infection with *L*. *infantum* significantly induced the development of IFN-γ-producing CD4^+^ T cells, in terms of both percentage (Fig 4C and 4D) and total cell number (Fig 4E), in both the WT and *Irf1*^−/−^ mice compared to their counterparts. However, it must be noted that the previously mentioned Th1 profile enhancement was heightened in the *Irf1*^−/−^ mice (Fig 4C–4E). The AST level was substantially increased in the infected *Irf1*^-/-^ mice (Fig 4F), indicating the presence of liver damage in these animals. Furthermore, based on surface marker analysis, the splenic DCs recovered from the infected *Irf1*^-/-^ mice presented slightly higher expression of MHCII, CD40 and CD86 than those from the infected WT mice (Fig 4G and 4H). Since it was demonstrated that IRF1 induces the expression of IFN-b and IL12 and binds to the IL12 gene promoter in peritoneal exudate cells after LPS or IFN-γ stimulation [41], we addressed whether the IL-12p40 levels are correlated with IRF1 expression. Thus, the expression of IL-12p40 in DCs from the spleen of infected *Irf1*^*-/-*^ mice was determined. The splenic DCs from infected *Irf1*^*-/-*^ mice presented increased expression of IL-12p40 compared with the WT DCs (Fig 4I), which demonstrates that IRF-1 downregulates IL-12 production by DCs during *L*. *infantum* infection. Together, the results indicate that IRF1 signaling through a TLR4-dependent pathway induces host susceptibility by downmodulating the Th1 response during *L*. *infantum* infection.

### The absence of IFNAR contributes to Th1 inflammation and parasite replication but is associated with immunopathology development

**Fig 5:**
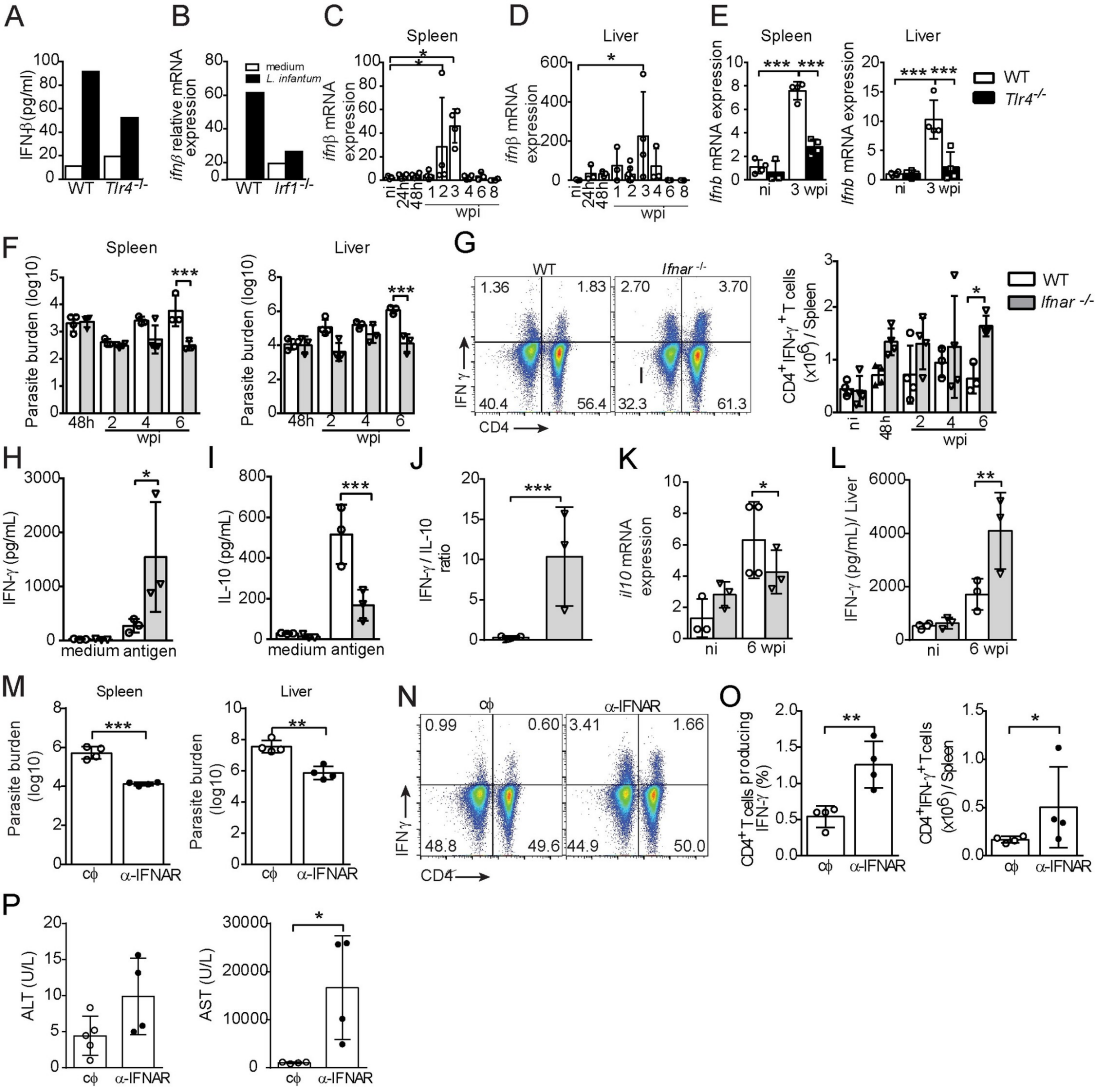
The absence of IFNAR contributes to Th1 inflammation and parasite replication but is associated with immunopathology development. (A) IFN-β levels in the supernatants of WT and *Tlr4*^-/-^ BMDCs. (B) mRNA expression of *Infβ* in WT and *Irf1*^-/-^ BMDCs stimulated with *L*. *infantum* or medium alone. (C and D) Kinetics of *Ifnβ* mRNA expression in the spleens (C) and livers (D) of C57BL/6-WT mice. (E) *Ifnb* mRNA expression in the spleens and livers of WT and *Tlr4*^*-/-*^ naïve and infected mice at 3 wpi. (F-L) WT and *Ifnar*^*-/-*^ mice were infected with 10^7^ *L*. *infantum* promastigotes. (F) Parasitic loads in the spleen and liver. (G) Representative dot plots of IFN-γ production by CD4^+^ T cells from the spleen in response to polyclonal restimulation at 6 wpi are shown, and the bar graphs represent the kinetics of the absolute number of IFN-γ-producing CD4^+^ T cells. (H) IFN-γ and (I) IL-10 levels in the supernatants of spleen cell cultures stimulated for 72 h with crude *L*. *infantum* antigen. (J) Ratio of IFN-γ/IL-10 production. (K) *Il10* mRNA expression in the liver. (L) IFN-γ levels in liver homogenate supernatants. (M-P) C57BL/6 WT mice were treated with an α-IFNAR antibody or an α-IgG control antibody, infected i.v. with 10^7^ *L*. *infantum* parasites and euthanized at 6 wpi. (M) Parasitic loads in the spleen and liver. (N) Representative dot plots of IFN-γ production by CD4^+^ T cells from the spleen in response to polyclonal restimulation. (O) Bar graphs showing the percentage and absolute number of IFN-γ-producing CD4^+^ T cells. (P) ALT and AST levels in serum. The data are expressed as the means ± SEMs (n = 3–5 mice in C-P or BMDCs assessed in quadruplicate in A and B) and are representative of three (A-L) or two (M-P) independent experiments. Statistical significance was calculated by one-way ANOVA with the Bonferroni post hoc test (A, B, C, D, E, F, G, H, I, K and L) or Student’s t test (J, M, O and P) (*p < 0.05, **p < 0.01, ***p < 0.001).

Because *Ifnb* mRNA was among the transcripts showing downregulated expression in the infected *Tlr4*^-/-^ BMDCs (Fig 2F), we evaluated whether IFN-β production is dependent on the triggering of the TLR4-IRF1 pathway during *in vitro L*. *infantum* infection. The infected BMDCs genetically deficient for TLR4 or IRF1 produced approximately two- to three-fold less IFN-β than the infected WT BMDCs (Fig 5A and 5B). The kinetics of IFN-β production in the spleen and liver at different time points after *in vivo* parasite infection were assessed by quantitative polymerase chain reaction (qPCR). Parasite infection induced significant mRNA expression in the spleen at 2 wpi and persisted for more than 3 wpi (Fig 5C), and significant IFN-β expression in the liver was detected at 3 wpi (Fig 5D). We evaluated the mRNA expression of *Ifnb* in infected *Tlr4*^*-/-*^ mice at 3 wpi, revealing a significant decrease in its expression in the spleens and livers of *Tlr4*^*-/-*^ mice (Fig 5E) at this time point, which demonstrates that IFN-β is produced during *in vivo L*. *infantum* infection and is dependent on TLR4.

Previous studies have shown that IFN-β inhibits the Th1 immune response during protozoan parasitic infections [42–45]. We thus investigated whether the IFNAR signaling could be responsible for development of the Th1 subset present in target organs of VL. To this end, we performed a kinetics analysis to monitor the infection and Th1 subset in infected mice with IFNAR deficiency (*Ifnar1*^-/-^) and control 129/SvEv WT mice. Among all the time points analyzed, the *Ifnar1*^-/-^ mice harbored fewer parasites in both target organs than the WT mice at 6 wpi (Fig 5F). In agreement with our above hypothesis, both the frequency and absolute number of IFN-γ-producing CD4^+^ T cells (Fig 5G) were significantly increased in the *Ifnar1*^-/-^ mice compared with their counterparts at this time point. Spleen cells from infected *Ifnar*^*-/-*^ mice restimulated with the crude antigen of *L*. *infantum* presented increased IFN-γ levels and decreased IL-10 levels compared with those in restimulated spleen cells from WT mice (Fig 5H and 5I, respectively). The ratio of IFN-γ/IL-10-producing cells demonstrated an enhancement of the Th1 response in *Ifnar*^*-/-*^ infected mice (Fig 5J). Strikingly, the transcript level of IL-10, an important anti-inflammatory cytokine that can be positively regulated by IFN-I [46,47], was reduced in the liver of the knockout mice at 6 wpi (Fig 5K). Indeed, the infected *Ifnar1*^-/-^ mice exhibited significantly increased IFN-γ levels in the liver compared with the infected WT mice (Fig 5L). Because 129/SvEv mice are considered genetically resistant to VL [48], we treated infected C57BL/6 mice with a specific α-IFNAR1 antibody to exclude the influence of the genetic background during *L*. *infantum* infection. In agreement with the phenotype of the infected *Ifnar1*^-/-^ mice, the α-IFNAR1 antibody treatment decreased the number of parasites in the spleen and liver (Fig 5M) and significantly increased both the percentage and absolute number of CD4^+^ T cells producing IFN-γ (Fig 5N and 5O) compared with the results obtained with IgG control treatment. These findings underscore the important role played by IFNAR in limiting CD4^+^IFN-γ^+^ T cells. As predicted based on the observation of Th1 inflammation, the AST level was greatly increased in the mice treated with the α-IFNAR1 antibody (Fig 5P). Taken together, our data demonstrate that IFN-I signaling restrains the Th1 immune response to *L*. *infantum* to favor parasite establishment but prevents liver injury.

### IFN-I limits the production of IFN-γ by Th1 cells

**Fig 6:**
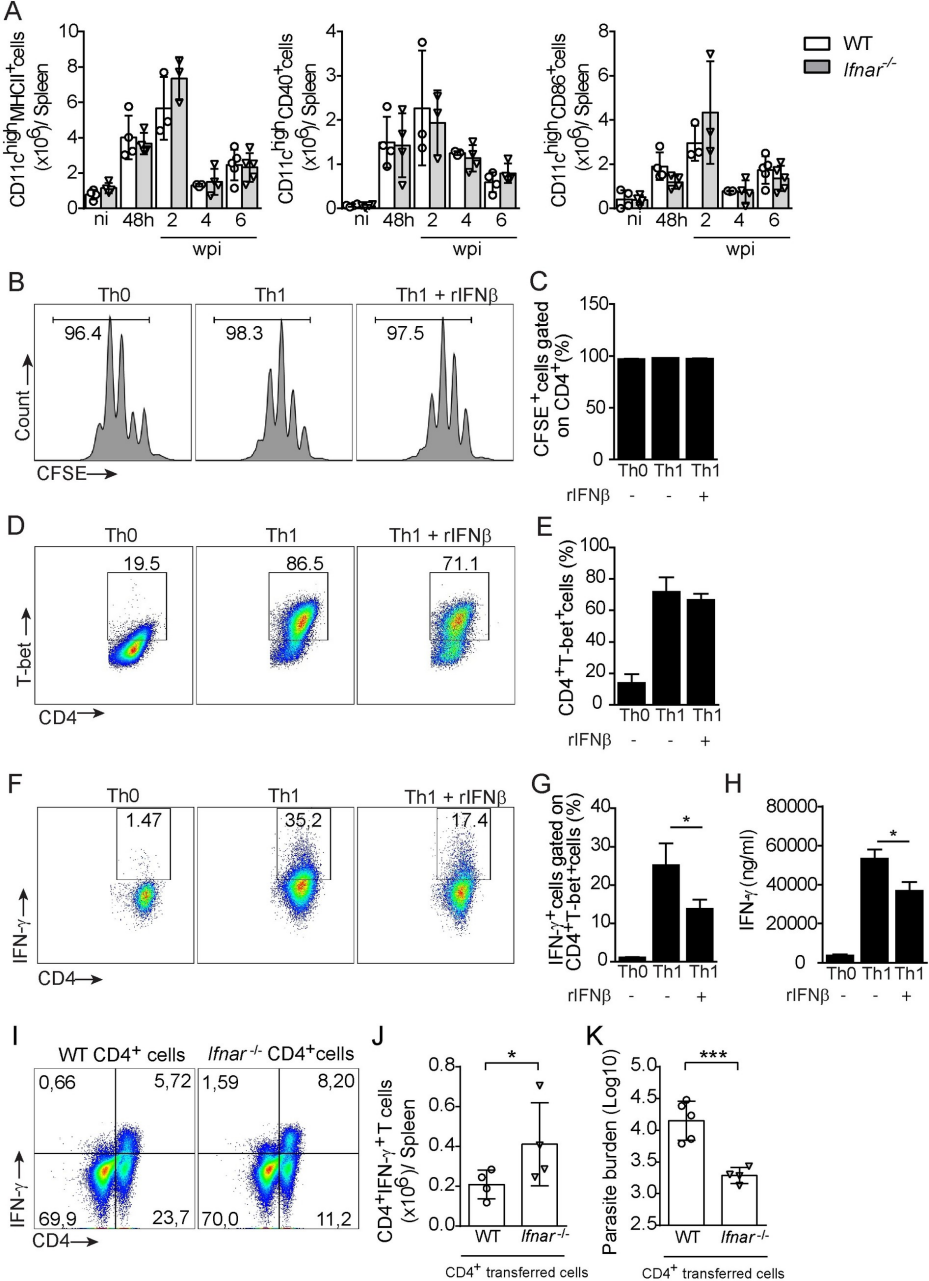
IFN-β limits Th1 responses independent of its suppressive effect on CD11b^+^ CD11c^high^ cells. (A) Absolute numbers of MHCII-, CD40- and CD86-expressing CD11b^+^ CD11c^high^ cells from the spleens of naïve and *L*. *infantum*-infected mice at the indicated time points after infection. (B-H) CD4^+^ cells from C57BL/6 WT naïve mice were cultured in the presence of α-CD3 and α-CD28 and differentiated into Th1 cells in the presence of recombinant IFN-β (1000 U/mL). (B) Representative histograms and (C) bar graph of CFSE-labeled CD4^+^ T cells. (D) Representative dot plots and (E) bar graph of the percentage of CD4^+^T-bet^+^ cells. (F) Representative dot plots and (G) bar graph of the percentage of CD4^+^IFN-γ^+^ cells within the T-bet^+^ cell population. (H) The IFN-γ levels in culture supernatants were measured by ELISA. (I-K) CD4^+^ cells from WT and *Ifnar*^*-/-*^ (C57BL/6 background) mice were transferred to *Rag1*^*-/-*^ mice (C57BL/6 background), and the Th1 response was evaluated at 6 wpi. (I) Representative dot plots of the percentage of CD4^+^IFN-γ^+^ cells. (J) Absolute number of CD4^+^IFN-γ^+^ cells. (K) Parasitic loads in the spleen. The data are expressed as the means ± SEMs (*in vitro* assay assessed in quadruplicate in B-H) and are representative of two independent experiments. Statistical significance was calculated by one-way ANOVA with the Bonferroni post hoc test (A, C, E, G and H) or Student’s t test (J and K) (*p < 0.05).

We subsequently performed functional assays to determine the relevance of IFN-I to the Th1 subset during VL. Because IFN-I signaling suppresses antiparasitic Th1 cells indirectly through DC modulation [42,49], we assessed the role of the early type I IFN response upon DC modulation by monitoring the kinetics of the expression of surface molecules related to DC activation, such as MHCII, CD40 and CD86, after *L*. *infantum* infection of WT and *Ifnar-/-* mice. No observable differences in the absolute numbers of CD11b^+^ CD11c^high^ cells expressing MHCII, CD40 and CD86 were found between infected *Ifnar1*^-/-^ and 129/SvEv WT mice (Fig 6A) at any of the time points analyzed, which suggests that Th1 commitment is not due to an effect of IFN-I on DC activation during infection.

We then examined whether IFN-I is involved in either T cell proliferation or Th1 generation. CD4 T cells were isolated from the spleens of naïve mice, labeled with carboxyfluorescein succinimidyl ester (CFSE), cultured under Th1 conditions in the presence or absence of recombinant IFN-β (rIFN-β) and then stimulated with α-CD3 and α-CD28 for 4 days. The proliferation of these cells was then analyzed by CFSE positivity, and Th1 differentiation was evaluated through intracellular staining of IFN-γ production or T-bet expression. In addition, the IFN-γ levels in the culture supernatant were measured. The addition of rIFN-β did not alter the frequency of CFSE-labeled CD4^+^ T cells **(**Fig 6B and 6C) or the expression of T-bet in CD4^+^ T cells (Fig 6D and 6E) but did reduced the frequency of IFN-γ^+^ cells among CD4^+^T-bet^+^ cells (Fig 6F and 6G) and the production of IFN-γ by Th1 cells (Fig 6H) by approximately 50% and 35%, respectively.

To address the role of IFNAR signaling specifically in T cells during *in vivo* infection, we transferred CD4^+^ cells from *Ifnar1*^-/-^ or C57BL/6 WT mice to Rag1-deficient mice (*Rag1*^*-/-*^ mice). The transferred mice were then infected, and the production of IFN-γ by their splenic CD4^+^ T cells was assessed at 6 wpi. Direct *ex vivo* flow cytometric measurement of IFN-γ production revealed that Rag mice transferred with *Ifnar1*^-/-^ CD4^+^ cells contained higher numbers of CD4^+^ T cells producing IFN-γ compared with those transferred with WT CD4^+^ cells (Fig 6I and 6J). Additionally, Rag mice transferred with *Ifnar1*^-/-^ CD4^+^ cells harbored significantly lower parasite loads in the spleen (Fig 6K). These data provide *in vivo* evidence showing that type I IFN signaling limits the production of IFN-γ specifically by Th1 cells. Together, these data demonstrate that IFNAR signaling directly affects the production of IFN-γ by Th1 cells.

### Asymptomatic subjects present a gene expression signature characterized by high TLR4 and IFN-I signaling

**Fig 7:**
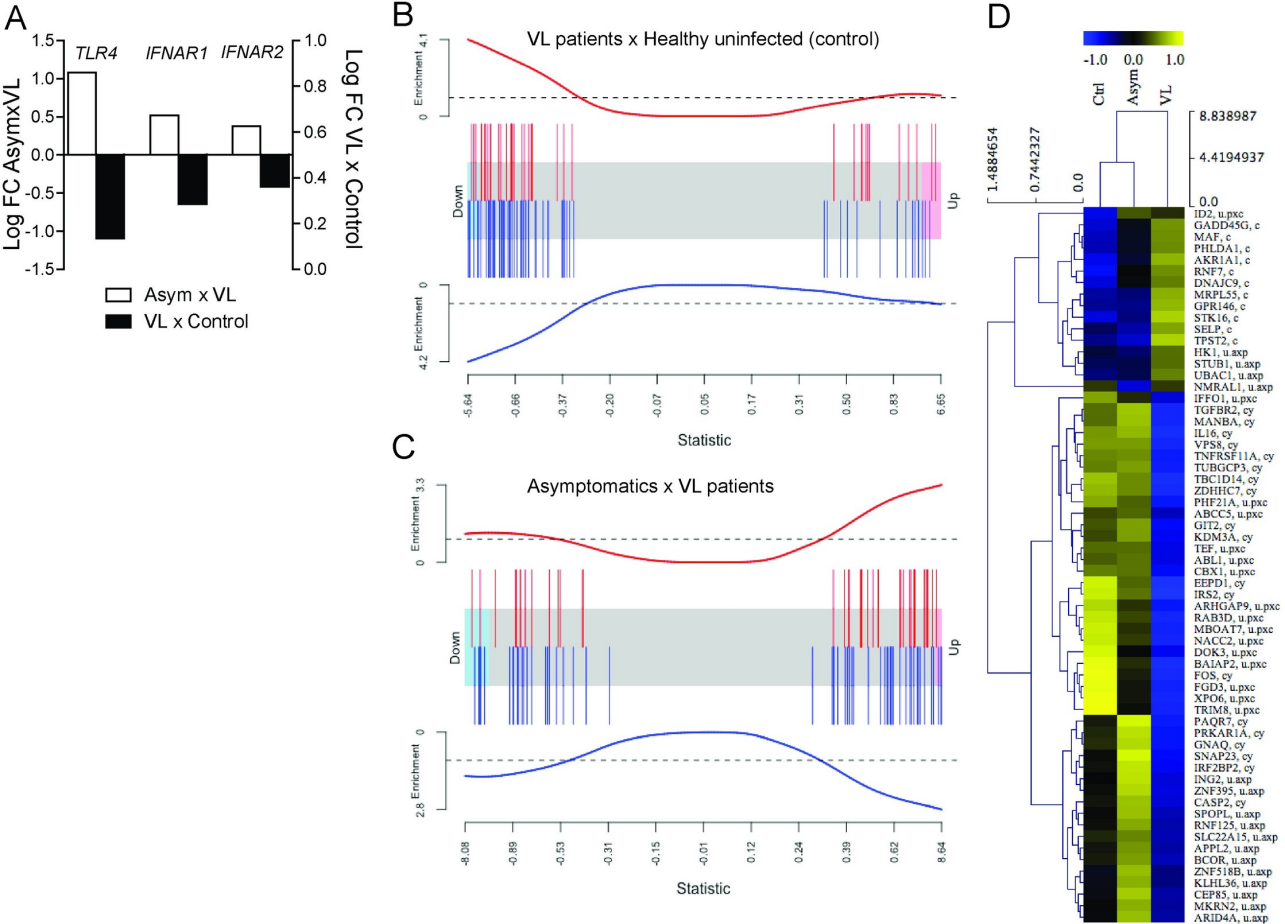
Peripheral blood mononuclear cells of asymptomatic subjects present a gene expression signature of high TLR4 and IFN-I signaling. (A) Fold change (log_2_FC) values for the *TLR4* (*false discovery rate (FDR) < 0.004), *IFNAR1* (**FDR < 0.02) *and IFNAR2* (***FDR < 0.05) genes in VL patients compared to healthy uninfected controls and asymptomatic subjects compared to VL patients. (B and C) Barcode plot of differential expression of gene set analysis using human macrophage gene signatures upon IFN-I stimulation (top panel in red) and human monocyte gene signatures upon TLR4 activation (bottom panel in blue). The red and blue worm lines represent the enrichment scores of the gene signature for IFN-I and TLR4 signaling. Each red and blue bar represents a differentially expressed gene at an FDR < 0.05, where down- and upregulated genes are placed on the left and right sides of the panel, respectively. (B) Gene set enrichment profile comparing VL patients to healthy uninfected controls. (C) Gene set enrichment profile comparing asymptomatic subjects to VL patients. (D) Heatmap of 62 differentially expressed genes (DEGs) related to gene signatures in IFN-I signaling (according to MSigDB) in the healthy uninfected control (Ctrl), asymptomatic (Asym) and VL groups. Plotted values used the average for each group, calculated from the z-score of logarithmic counts per million reads of normalized RNA sequencing libraries. The color scale bar indicates downregulated (blue) or upregulated (yellow) genes. The statistical significance of differentially expressed genes was calculated using p < 0.05 adjusted to an FDR < 0.05. “c” indicates common genes between VL patients and healthy uninfected controls and between asymptomatic subjects and VL patients; “u.pxc” and “u.axp” indicate genes exclusively found in VL patients *versus* healthy uninfected controls and in asymptomatic subjects *versus* VL patients, respectively; “cy” indicates common genes between both comparisons and significantly enriched in the gene set test (p < 0.05, *mroast* function of edgeR package).

Patients who develop severe clinical manifestations present a “cytokine storm” characterized by high systemic levels of pro- and anti-inflammatory mediators [50,51]. Nonetheless, individuals exhibiting high systemic levels of IFN-γ and expression of regulatory cytokines exhibit no disease progression [52], which suggests that a lack of regulation of the immune response is a determining factor in the development of severe forms of VL. To evaluate the TLR4-IFN-I pathway in human VL samples, an RNA sequence analysis of total leukocyte samples from healthy control donors, asymptomatic individuals and VL patients was performed. The cytokine storm status of VL patients included in this work was previously published by dos Santos et al., who demonstrated that VL patients present higher levels of cytokines compared with healthy controls and DTH^+^ individuals [8]. The gene expression levels demonstrated that *tlr4*, *ifnar1* and *ifnar2* expression was induced in asymptomatic subjects and negatively regulated in VL patients (Fig 7A).

An enrichment analysis of genes related to the TLR4 and IFN-I pathways was performed based on a collection of immunological signatures (ImmuneSigDB). The comparison of the VL patients with the control subjects revealed that many transcripts related to the IFN-I (red bars) and TLR4 (blue bars) pathways were positively modulated in the asymptomatic patients and negatively modulated in the VL patients (Fig 7B). In agreement with this finding, the comparison of the asymptomatic subjects with the VL patients showed that many transcripts related to the IFN-I (red bars) and TLR4 (blue bars) pathways were downregulated in the VL patients (Fig 7B) and upregulated in the asymptomatic subjects (Fig 7C).

In each comparison, a total of 46 differentially expressed genes (DEGs) matched the gene signature list for the IFN-I signaling pathway. By overlapping these DEGs (red bars in Fig 7B and 7C), we found that 30 were common to both comparisons (genes indicated as “c” in Fig 7D) and 16 were unique (genes indicated as “u.pxc” or “u.axp” in Fig 7D), and all of these DEGs presented opposite expression profiles in the VL patient group compared with the nonsick groups (asymptomatic and control groups, Fig 7D). Out of the 30 common DEGs, 19 were significantly enriched in the test gene set (genes indicated as “cy” in Fig 7D). Together, these data demonstrate that TLR4 and IFN-I are related to the prevention of the severe form of VL.

## Discussion

The magnitude of immune response activation against chronic infections must be properly regulated to provide maximal protection from infection with minimal tissue lesion. In this sense, the innate immune mechanisms can fine-tune the subsequent adaptive immune response following the recognition of microorganisms and influence the nature and magnitude of this subsequent response [53]. Our group has made recent progress in understanding the role of TLRs in VL. While TLR2 and TLR9 promote the development of the immune response to ensure parasite control [20,21], the present study demonstrated that TLR4 acts to limit the parasite-specific immune response. According to our results, the TLR4 pathway negatively regulates the immune response to *L*. *infantum* infection through IRF1, culminating in IFN-β production, which in turn dampens Th1 cell functions to avoid immunopathology development. This signaling pathway could be associated with the prevention of severe forms of disease in humans since the asymptomatic subjects presented upregulation of the expression of TLR4- and IFN-I-related signaling genes, which agrees with the experimental model data.

TLRs can promote anti-inflammatory responses for homeostasis maintenance [54,55]. Although the proinflammatory effects of TLR4 signaling have been described in detail, the anti-inflammatory responses induced by TLR4 activation are much less characterized. According to our data, BMDCs with a TRIF genetic deficiency infected with *L*. *infantum* produce higher levels of IL-12p40, while those with a MyD88 deficiency have completely lost the ability to secrete IL-12p40. In fact, the cell compartment where TLR4 signaling occurs seems to be an important factor in deciding the fate of pro- and anti-inflammatory signaling [56]. When TLR4 signaling occurs in the endosomal vesicle, the TLR4-ligand complex recruits the TRIF adapter molecule that induces the secretion of IFN-I and anti-inflammatory cytokines such as IL-10 in a manner dependent on IRFs and NF-κB [56].

To identify the sequential order of the pathways involved in the TLR4-dependent pathway, we explored the transcriptional patterns of genes related to TLR signaling in WT and *Tlr4*^-/-^ BMDCs infected with *L*. *infantum*. *In vitro* infection of DCs induces a slight upregulation of transcript expression, reinforcing the idea that *L*. *infantum* can silently survive inside DCs [16,17]. We observed that IRF1 expression is downstream of TLR4 in *in vitro* BMDCs infected with parasites and in target organs during *in vivo L*. *infantum* infection. TLR4 signaling triggers members of the IRF transcription factor family, which in turn play an important role in the regulation of the transcription of immune system genes [57]. Among the family of IRF transcription factors, IRF1 was the first member identified [58] and was initially characterized by the ability to induce the transcription of IFN-β [58].

Murray et al. indicated that TLR4 restrains the spread of *L*. *donovani*, while TLR2 signaling downregulates macrophage antileishmanial activity. We previously demonstrated that TLR2 exerts a protective effect during *L*. *infantum* infection [59], but the present work shows that TLR4 signaling halts an ongoing protective immune response. It is possible that these contrasting results are due to the parasite species because several molecules show distinct expression between these species [60]. In addition, we cannot exclude that the parasitic stage triggers distinct roles of TLRs in viscerotropic diseases. Between 24 and 48 h after parasite internalization, promastigotes transform into their nonflagellated amastigote forms, and this transformation has been associated with the upregulation of Th2-related cytokines [61]. Another study compared the infection course of *Phlebotomus argentipes* females obtained with promastigotes of *L*. *donovani* with that observed with the amastigote form of this parasite. Early-stage infections were associated with substantial differences in both the parasitic load and the representation of morphological forms, and these differences gradually disappeared with increasing maturation of the infectious agent. This finding shows that use of the promastigote stage for sandfly infection does not significantly alter the final outcome of *Leishmania donovani* development in *P*. *argentipes* or the resulting transmissibility [62].

Our results demonstrated that IFN-β secretion is dependent on the TLR4-IRF1 pathway, which in turn directly mitigates Th1 immune responses. Indeed, IFNAR deficiency and specific IFNAR blockade improve the Th1 response to *L*. *infantum* infection and, as a consequence of the exacerbated immune response, increase the level of serum transaminases. Cellular responses to IFNAR signaling are dependent on the cell type and vary during the course of an immune response in a context-dependent manner. Pathogens and immune and environmental factors modulate IFNAR signaling and IFN-stimulated gene (ISG) expression [63]. Our data demonstrate that IFNAR directly affects the production of IFN-γ by Th1 cells during chronic VL infection. The cell responses to IFNAR ligation can be regulated at several levels: the expression and posttranslational modification of IFNAR signaling and the induction of transcription factors that interfere with gene expression [64]. The role of IFNAR signaling can be distinct in the context of other parasitic infections; for example, in *P*. *chabaudi*-infected mice, IFN-I suppresses Th1 responses by modulating DC functions [42]. In contrast, treatment with HuIFN-β showed a protective effect, which was enhanced by the combination of rHuIFN-β and LPS and was IFN-γ dependent in the model of toxoplasmosis [65]. In addition, *Ifnar*^-/-^ mice infected with *T*. *gondii* presented an increased parasitic load, and higher parasitic burdens correlated with decreased mouse survival [66], suggesting that IFN-β may be produced at the onset of infection to enhance IFN-γ. Interestingly, during *T*. *brucei* infection, type I IFNs play a role in the early control of parasites but contribute to the downregulation of IFN-γ production in the chronic phase of infection [67].

The modulatory function of IFN-I may be dependent upon the timing of production and upon specific pathogens, as IFNAR signaling assumes an immunosuppressive role to limit host toxicity when the microorganism cannot be cleared [64]. We observed that the delayed kinetics of IFN-β expression (at 3 wpi) affected the TLR4-mediated impact on DCs, interfering with the Th1 response and parasite control during the chronic phases of infection. In a malaria model, the production of IFN-I promotes the innate and adaptive immune responses, while delayed IFN-I production impairs both immune responses [68]. IFN-I may have a predominantly immunomodulatory role during the chronic phase of infection. IFN-I induces the expression of suppressive factors, such as IL-10 and PD-L1, in DCs and induces the expression of proinflammatory cytokines during lymphocytic choriomeningitis virus (LCMV) infection. Suppressive factors play a dominant role since their absence decreases the expression of IL-10 and PDL1 and promotes viral clearance in an IFN-γ-producing CD4^+^ T-cell-dependent manner [43,69]. During *Mycobacterium tuberculosis* and *Mycobacterium leprae* infections, IFN-I suppresses Th1 cells through the induction of IL-10 expression [44,46]. In addition, IFNAR blockade during the chronic phase of human immunodeficiency virus (HIV) infection enhances CD8^+^ T-cell activation and reduces the viral burden in the blood [70,71]. As VL generates chronic inflammation through the persistence of the parasite, based on our findings, we propose that IFN-β assumes a predominantly immunomodulatory role to limit this undesired side effect.

Gene expression analyses of human VL demonstrated upregulation and downregulation of the expression of transcripts related to the TLR4-IFN-I pathway in asymptomatic subjects and VL patients, respectively, and thus provide direct evidence showing that the TLR4-IFN-I pathway is related to the asymptomatic form. Indeed, patients with the severe form of VL develop a clinical condition known as cytokine storm, which is characterized by elevated serum levels of pro- and anti-inflammatory cytokines that return to baseline levels after the chemotherapeutic process [50,72,73]. Cytokine storm is considered the direct cause of the severe form of human VL. The intense release of inflammatory mediators is clinically important, as it determines the severity of disease through the tissue damage [74], suggesting that there is a failure in the regulatory mechanisms of these subjects. IFN-I signaling is upstream of hundreds of proinflammatory and anti-inflammatory genes [75,76]. Interestingly, the gene signature for IFN-I demonstrates an upregulation of the expression of transcripts related to the negative regulation of the immune response process in asymptomatic and control subjects; in contrast, the expression of these genes is downregulated in VL patients. Among these transcripts, we highlight the *MKRN2* gene, which is a p65 ubiquitin E3 ligase that binds to the p65 subunit and promotes proteasome-dependent degradation, thereby suppressing p65-mediated NFκB transactivation [77]; *RNF125*, which encodes an E3 ubiquitin ligase that acts as a positive regulator in the T-cell receptor signaling pathway [78,79]; *DOK3*, which encodes a novel adapter molecule, DOK3, which is highly expressed in hematopoietic cells, that is involved in the inhibition of immunoreceptor-mediated nuclear factor of activated T-cell (NFAT) activation and cytokine release [80] and that negatively regulates TLR signaling [81]; and the *NACC2* gene, which is a transcriptional repressor and an important regulator of the p53 pathway [82]. Given this information, we believe that there is a failure in immune regulation in VL patients, which in turn leads to the development of a cytokine storm and consequently severe disease.

**Fig 8:**
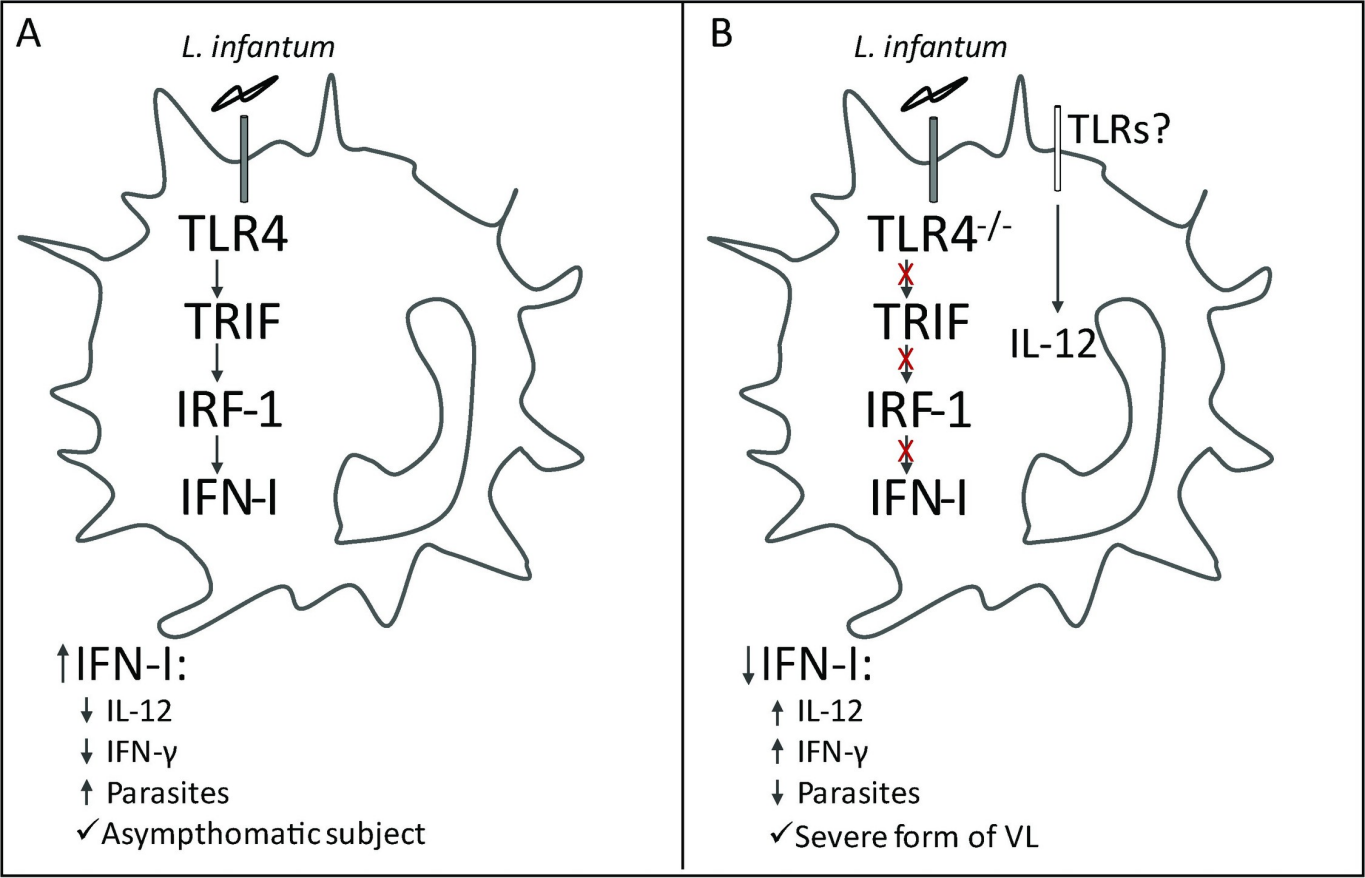
The TLR4-IRF1 pathway culminates in IFN-β production to restrict the Th1 immune response in visceral leishmaniasis. (A) TLR4 through TRIF signaling leads to IRF1 activation and IFN-I production, and these effects hamper the Th1 response to prevent immunopathology during L. infantum infection. (B) The absence of TLR4 signaling and IFN-I production contributes to the Th1 response and restricts parasite replication but causes immunopathology. In humans, the TLR4 and IFN-I pathways are positively modulated in asymptomatic subjects compared with VL patients, and this finding provides direct evidence that TLR4-IFN-I signaling is related to the prevention of the severe form of VL.

Taken together, our results indicate that TLR4 and IRF1 signaling culminate in the production of IFN-β, ensuing the regulation of the immune response against *L*. *infantum* infection and in turn avoiding an exacerbated immune response and the consequent immunopathology (Fig 8). Thus, our findings provide a potential therapeutic target to treat or prevent the clinical complications of VL.

## Materials and methods

### Animals

Six- to 8-week-old mice were used for the infection experiments. Female C57BL/6 (wild type—WT), C57BL/6 *Tlr4*^-/-^, C57BL/6 *Trif*^-/-^, C57BL/6 *Myd88*^-/-^, C57BL/6 *Ifnar*^-/^, C57BL/6 *Rag1*^*-/-*^, C57BL/6 *Cd14*^*-/-*^, C57BL/6 *Irf3*^*-/-*^, 129/SvEv (wild type—WT), 129/SvEv *Irf1*^-/-^ and 129/SvEv *Ifnar*^-/-^ mice were obtained from the animal facilities at the School of Medicine of Ribeirão Preto, University of São Paulo (Brazil). The mice were housed in temperature-controlled rooms (22–25°C) at the animal facility of the Department of Biochemistry and Immunology (School of Medicine of Ribeirão Preto) and received water and food *ad libitum*. All the experiments were conducted in accordance with the National Institutes of Health (NIH) guidelines for the welfare of experimental animals and with approval from the Ethics Committee of the School of Medicine of Ribeirão Preto.

### Parasite culture, infection and load estimation

*L*. *infantum* (isolate HU-UFS14) was cultured in Schneider medium with 20% heat-inactivated fetal bovine serum (FBS), 5% penicillin and streptomycin (both from Sigma-Aldrich, St. Louis, MO, USA) and 2% male human urine. The parasite virulence was maintained by serial passaging in BALB/c mice and by culturing *in vitro* for no more than five passages. The mice were intravenously infected via the retro-orbital plexus with 10^7^ stationary-phase *L*. *infantum* promastigotes in 100 μL of phosphate-buffered saline (PBS). The hepatic and splenic parasite burdens were determined using a quantitative limiting dilution assay.

### In vivo α-IFNAR antibody treatment

C57BL/6 mice were treated intraperitoneally with 100 μg of α-mouse IFNAR antibody (BioXCell, West Lebanon, NH, USA, MAR1-5A3 clone) in PBS 1 day prior to infection and 50 μg of antibody once a week until 6 wpi. A corresponding IgG isotype antibody served as a control (Miltenyi Biotec).

### DC generation and infection

The femurs of naïve mice were washed with medium, and BMDCs were generated over 6 days using complete RPMI 1640 medium supplemented with 20 ng/mL GM-CSF (PeproTech, cat. number 315–03), as previously described [83]. For the DC activation assay, BMDCs (1 × 10^6^ cells/mL) cultured in RPMI 1640 medium supplemented with 10% FBS were infected with *L*. *infantum* promastigotes at a 1:5 ratio (cells:parasites). BMDCs were also treated with LPS (InvivoGen, cat. number #tlrl-peklps) at 200 ng/mL.

### Splenocyte cell culture

Single-cell suspensions were aseptically prepared from spleen tissue samples, diluted to a concentration of 2 × 10^6^ cells/mL, seeded into 48-well plates in a total volume of 500 μL of complete RPMI 1640 medium (1 × 10^6^ cells/well; Gibco) and stimulated with *L*. *infantum* crude antigen (50 μg/mL). The cell culture supernatants were harvested after 72 h of culture at 37°C in 5% CO_2_.

### Th1 differentiation and culture

For *in vitro* Th1 cell differentiation, isolated CD4^+^ cells (MACS Miltenyi Biotec) were stimulated with plate-bound α-CD3 (2 μg/mL) and α-CD28 (1 μg/mL) (BioLegend, 17A2 and 37.51 clone, respectively). The experimental Th1 conditions also included the use of rMuIL-12 (5 ng/mL), IL-2 (25 U/mL), and α-IL-4 antibodies (10 μg/mL) in the presence or absence of rMuIFN-β (1000 U/mL) for 96 h. The cells were labeled with CFSE for the proliferation assay and stained for CD4, T-bet and IFN-γ expression. The supernatants were collected for measurement of the IFN-γ level by enzyme-linked immunosorbent assay (ELISA). All recombinant cytokines were obtained from R&D Systems (Minneapolis, MN, USA).

### Quantification of cytokines

The concentrations of IFN-γ and IL-10 in the supernatants of restimulated splenocytes were determined using commercial ELISA kits. For the detection of IFN-γ in the liver, tissue samples were weighed and titrated in 1 mL of PBS Complete (Roche Diagnostics, Mannheim, Germany) containing a protease inhibitor cocktail. For IFN-β quantification, the supernatant from stimulated BMDCs was recovered after 24 h of stimulation. The levels of cytokines were determined using commercial ELISA kits for IFN-γ and IL-10 (both obtained from BD Biosciences) and for IFN-β (obtained from PBL Assay Science). Wavelength correction and background signals were subtracted from the absorbance values.

### Flow cytometry assay

For intracellular staining, the cells were cultured with PMA (50 ng/mL) and ionomycin (500 ng/mL; Sigma-Aldrich, St Louis, MO, USA) as well as brefeldin A (BioLegend, San Diego, CA, USA) for 4 h. The cells were then fixed with 4% paraformaldehyde and permeabilized with 0.5% saponin. Cell acquisition was performed using a FACSort flow cytometer. The data were plotted and analyzed using FlowJo software (TreeStar, Ashland, OR, USA). The antibodies used are listed in the Supporting Protocol.

### Quantitative real-time PCR

The protocols used for total RNA isolation and mRNA expression analysis by quantitative real-time PCR (qPCR) are detailed in the Supplemental Methods section. The primers used in qPCR are listed in S1 Table.

### Adoptive transfer of CD4^+^ cells

Leukocytes were isolated from the spleen and lymph nodes and incubated with anti-CD4 magnetic beads (5 μL/10^7^ cells; Miltenyi Biotec, Surrey, U.K.) for 15 min at 4°C. The cells were washed, resuspended at 10^8^ cells/mL, and positively selected on a MACS column (Miltenyi Biotec). The purification of the cells was assessed by FACS using anti-CD4 (RM4-5) and anti-CD62L (MEL-14) antibodies. A total of 5 x 10^6^ CD4^+^ cells derived from WT or *Ifnar*^*-/-*^ mice (C57BL/6 background) were transferred to C57BL/6 *Rag1*^*-/-*^ mice in 100 μL of PBS. All the mice were infected with *L*. *infantum* 1 day after transfer.

### Patients

Human PBMCs were obtained from Hospital Universitário from Universidade Federal de Sergipe. The Ethics Committee of the Federal University of Sergipe approved these procedures. The subjects or their legal guardians signed an informed consent form. Ethical approval was obtained from the Hospital Universitário from Universidade Federal de Sergipe, Comissão Nacional de Ética em Pesquisa (CONEP), CAAE 0151.0.107.000–07 and CAAE 0123.0.107.000–11.

### Statistical analysis

The analyses were performed using Prism 5.0 software (GraphPad Software, Inc.). Each variable from data collected in the experiments was subjected to analysis of normal distribution and homogeneity of variance. Parametric analyses such as Student’s *t* test and analysis of variance (ANOVA) were applied to data presenting with normal distribution and variance homogeneity. The means obtained for the different groups were compared by one-way ANOVA followed by the Bonferroni post hoc test. Comparisons between two sample groups were performed using unpaired two-tailed Student’s *t* test. The data are expressed as the means ± SEMs and are representative of two to four independent experiments. Differences were considered significant at *p < 0.05, **p < 0.01, and ***p < 0.001.

## Supporting information

S1 Fig Gating strategy used to define the leukocyte population. (A) The cells were gated based on their forward scatter height (FSC) and area (SSC) within the areas containing the populations of lymphocytes (G1) and myeloid cells (G2). (B) Lymphocytes (G1) were identified as B cells (CD19^+^ cells, G3) or T cells (CD3^+^ cells, G4). (C) The myeloid cell gate (G2) was obtained by first identifying CD11b^+^ cells (G5) and then (D) identifying dendritic cells (CD11c^high^ cells, G6) and macrophages (F4/80^+^ cells, G7).
(TIF)

S2 Fig Dendritic cells are the major cellular subset to express TLR4 during *L*. *infantum* infection. Percentage and absolute number of TLR4 expression in the populations of CD11b^+^ CD11c^high^ cells (A and B), CD11b^+^ F4/80^+^ cells (C and D), CD3^+^ cells (E and F) and CD19^+^ cells (G and H) from naïve and *L*. *infantum*-infected C57BL/6 WT mice at the indicated time points after infection. The data are expressed as the means ± SEMs (n = 4 mice). The statistical significance was calculated by one-way ANOVA with the Bonferroni post hoc test (*p < 0.05, **p < 0.01, and ***p < 0.001).
(TIF)

S3 Fig CD14 does not participate during *L*. *infantum* infection. (A-H) WT and *Cd14*^*-*/-^ mice were i.v. infected with 10^7^ *L*. *infantum* parasites and euthanized at 6 wpi. (A) Representative histograms of MHCII and CD86 expression in CD11b^+^CD11c^high^ cells from the spleen of *L*. *infantum*-infected mice. (B and C) Absolute number of MHCII- and CD86-expressing splenic CD11b^+^CD11c^high^ cells. (D) Representative dot plots showing the production of IFN-γ by CD4^+^ T cells from the spleen in response to polyclonal restimulation. (E-F) Graph bars representing the percentage (E) and absolute number (F) of IFN-γ-producing CD4^+^ T cells in the spleen. (H) Parasite loads in the spleen and liver. The data are expressed as the means ± SEMs (n = 4 mice). The statistical significance was calculated by one-way ANOVA with the Bonferroni post hoc test (B, C, E and F) or Student’s t test (H).
(TIF)

S4 Fig Costimulatory molecules are not altered in splenic DCs from *Tlr4*^*-/-*^ infected mice. WT and *Tlr4*^*-*/-^ mice were i.v. infected with 10^7^ *L*. *infantum* parasites and euthanized at 6 wpi. (A and D) Representative histograms of PD-L1 and CTLA-4 expression in the population of CD11b^+^CD11c^high^ cells from the spleen of *L*. *infantum*-infected mice. (B and E) Percentage and (C and F) MFI of PD-L1 and CTLA-4 obtained for the population of splenic CD11b^+^CD11c^high^ cells from naïve and *L*. *infantum*-infected mice. The data are expressed as the means ± SEMs (n = 4–5 mice). The statistical significance was calculated by one-way ANOVA with the Bonferroni post hoc test (B, C, E and F).
(TIF)

S5 Fig IRF3 contributes to the activation of DCs during *L*. *infantum* infection. WT and *Irf3*^*-/-*^ BMDCs were infected with *L*. *infantum* (5 parasites:1 cell) or not infected (medium) for 24 h. Graph bars of the MFI of MHCII (A) and CD86 (B) are shown. (C) Representative dot plots showing the production of IL-12p40 by CD11c^+^ cells. (D) Graph bars showing the percentage of CD11c^+^ IL-12p40^+^ cells. (E) The IL-12p40 levels in culture supernatants were measured by ELISA. The data are expressed as the means ± SEMs (BMDCs were assessed in quadruplicate). The statistical significance was calculated by one-way ANOVA with the Bonferroni post hoc test (*p < 0.05 and ***p < 0.001).
(TIF)

S1 Table Differentially expressed (DE) genes. (XLSX)

S1 Protocol Supporting protocol. (DOCX)
