# Supplementary figures and images for: TLR4 abrogates the Th1 immune response through IRF1 and IFN-β to prevent immunopathology during L. infantum infection
Source: PLoS Pathog. 2020 Mar 25;16(3):e1008435. doi: 10.1371/journal.ppat.1008435 (PMC7135367; doi:10.1371/journal.ppat.1008435)

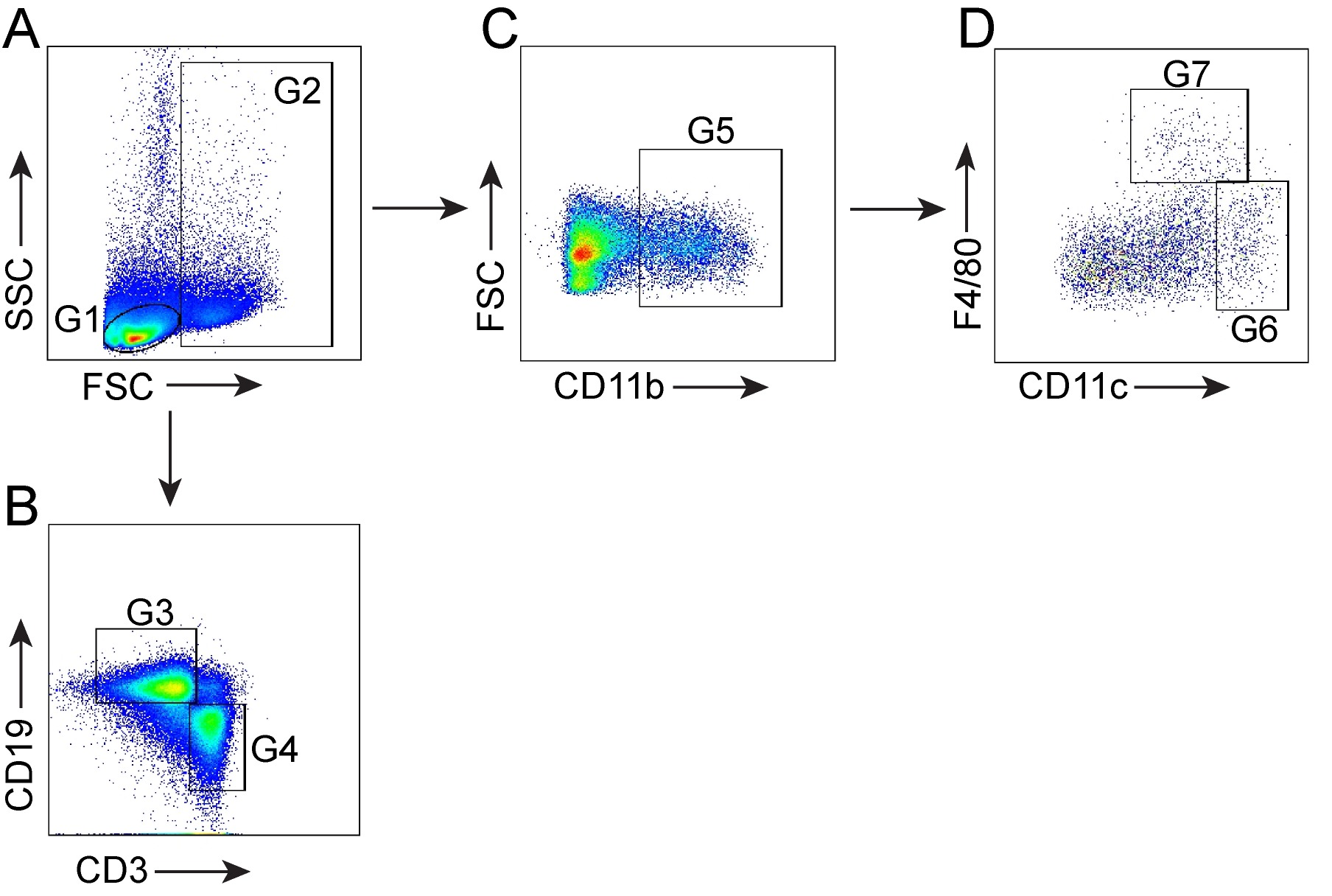

Supplement: S1 Fig — (A) The cells were gated based on their forward scatter height (FSC) and area (SSC) within the areas containing the populations of lymphocytes (G1) and myeloid cells (G2). (B) Lymphocytes (G1) were identified as B cells (CD19+ cells, G3) or T cells (CD3+ cells, G4). (C) The myeloid cell gate (G2) was obtained by first identifying CD11b+ cells (G5) and then (D) identifying dendritic cells (CD11chigh cells, G6) and macrophages (F4/80+ cells, G7). (TIF) [file ppat.1008435.s001.tif]

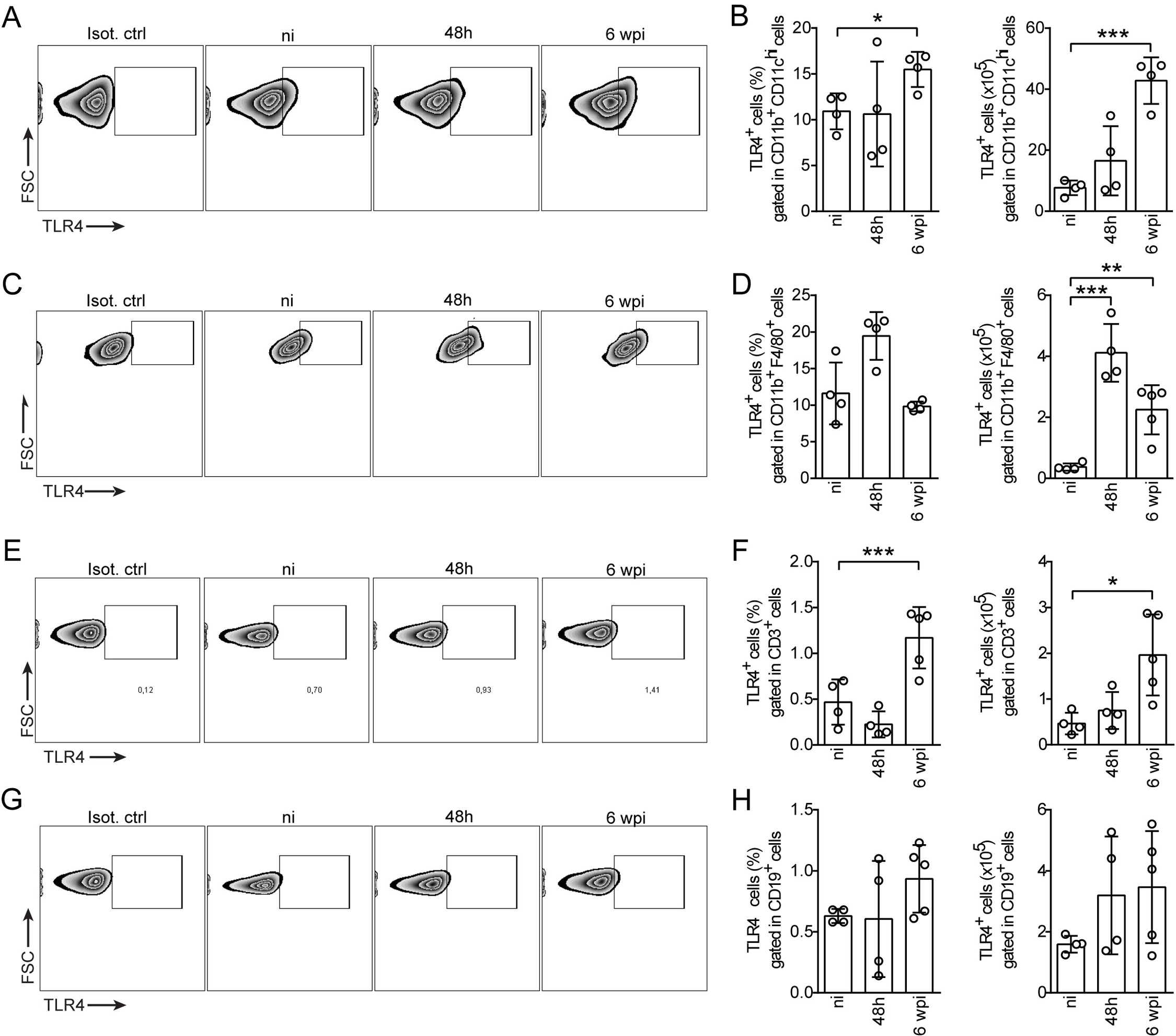

Supplement: S2 Fig — Percentage and absolute number of TLR4 expression in the populations of CD11b+ CD11chigh cells (A and B), CD11b+ F4/80+ cells (C and D), CD3+ cells (E and F) and CD19+ cells (G and H) from naïve and L. infantum-infected C57BL/6 WT mice at the indicated time points after infection. The data are expressed as the means ± SEMs (n = 4 mice). The statistical significance was calculated by one-way ANOVA with the Bonferroni post hoc test (*p < 0.05, **p < 0.01, and ***p < 0.001). (TIF) [file ppat.1008435.s002.tif]

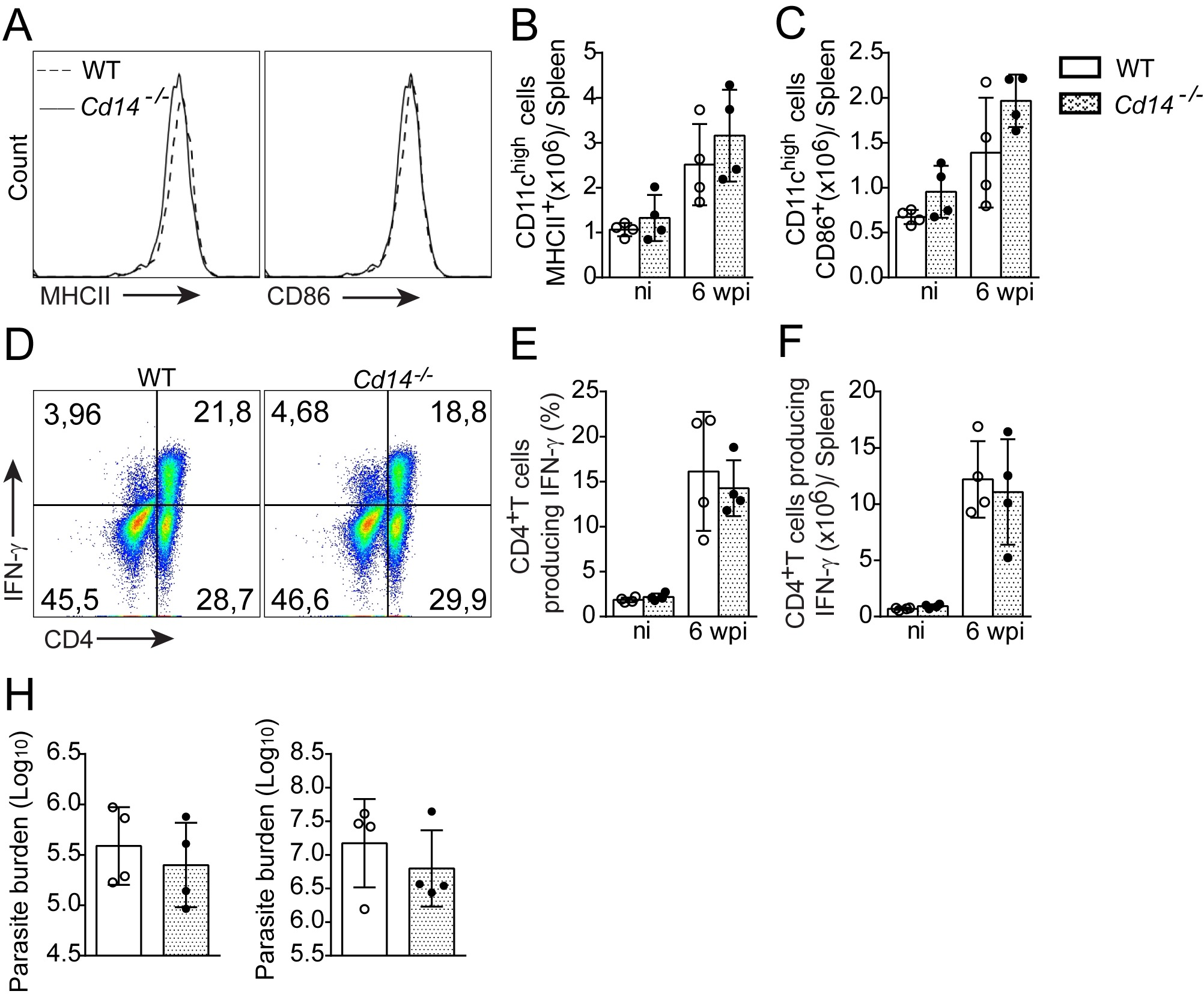

Supplement: S3 Fig — (A-H) WT and Cd14-/- mice were i.v. infected with 107 L. infantum parasites and euthanized at 6 wpi. (A) Representative histograms of MHCII and CD86 expression in CD11b+CD11chigh cells from the spleen of L. infantum-infected mice. (B and C) Absolute number of MHCII- and CD86-expressing splenic CD11b+CD11chigh cells. (D) Representative dot plots showing the production of IFN-γ by CD4+ T cells from the spleen in response to polyclonal restimulation. (E-F) Graph bars representing the percentage (E) and absolute number (F) of IFN-γ-producing CD4+ T cells in the spleen. (H) Parasite loads in the spleen and liver. The data are expressed as the means ± SEMs (n = 4 mice). The statistical significance was calculated by one-way ANOVA with the Bonferroni post hoc test (B, C, E and F) or Student’s t test (H). (TIF) [file ppat.1008435.s003.tif]

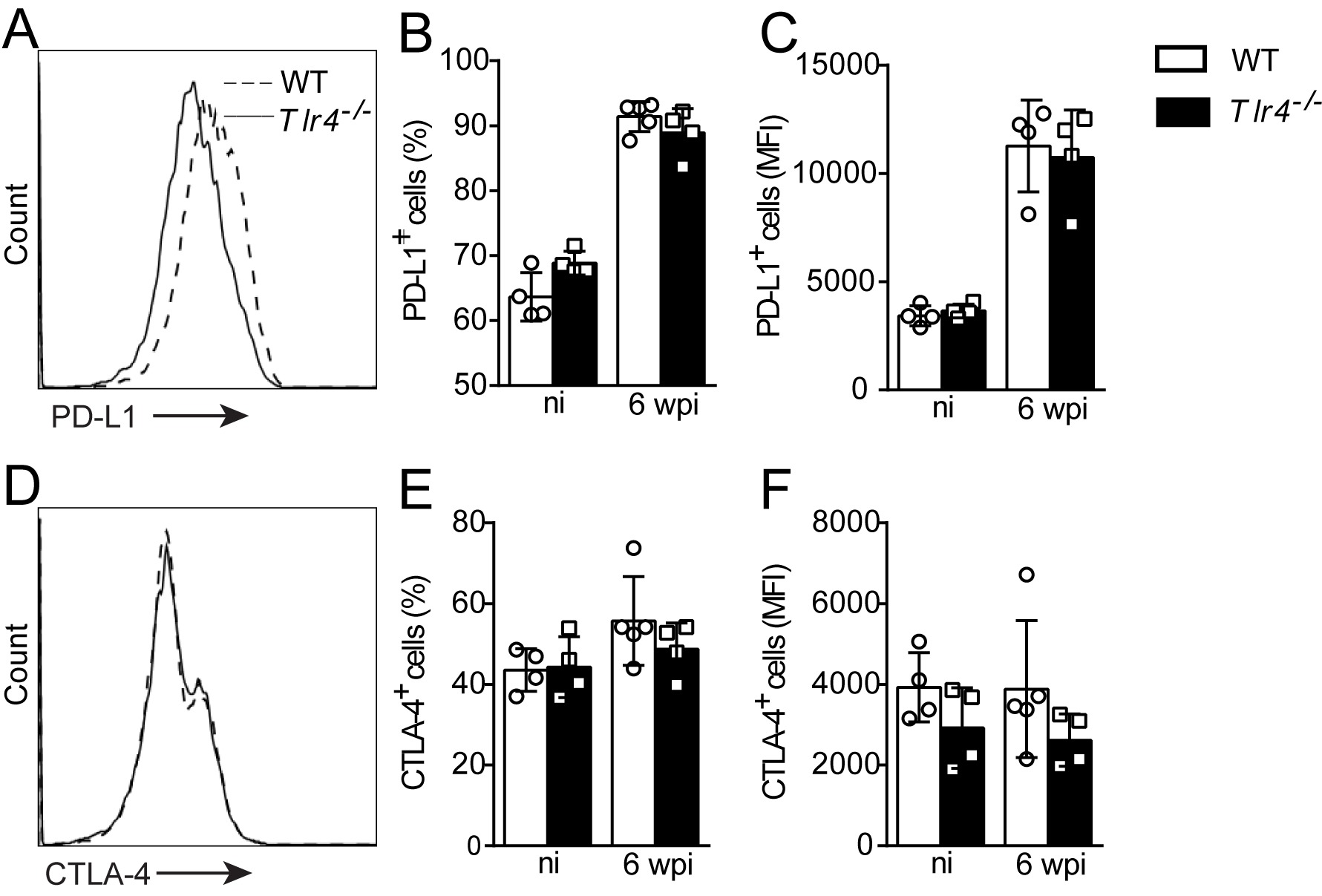

Supplement: S4 Fig — WT and Tlr4-/- mice were i.v. infected with 107 L. infantum parasites and euthanized at 6 wpi. (A and D) Representative histograms of PD-L1 and CTLA-4 expression in the population of CD11b+CD11chigh cells from the spleen of L. infantum-infected mice. (B and E) Percentage and (C and F) MFI of PD-L1 and CTLA-4 obtained for the population of splenic CD11b+CD11chigh cells from naïve and L. infantum-infected mice. The data are expressed as the means ± SEMs (n = 4–5 mice). The statistical significance was calculated by one-way ANOVA with the Bonferroni post hoc test (B, C, E and F). (TIF) [file ppat.1008435.s004.tif]

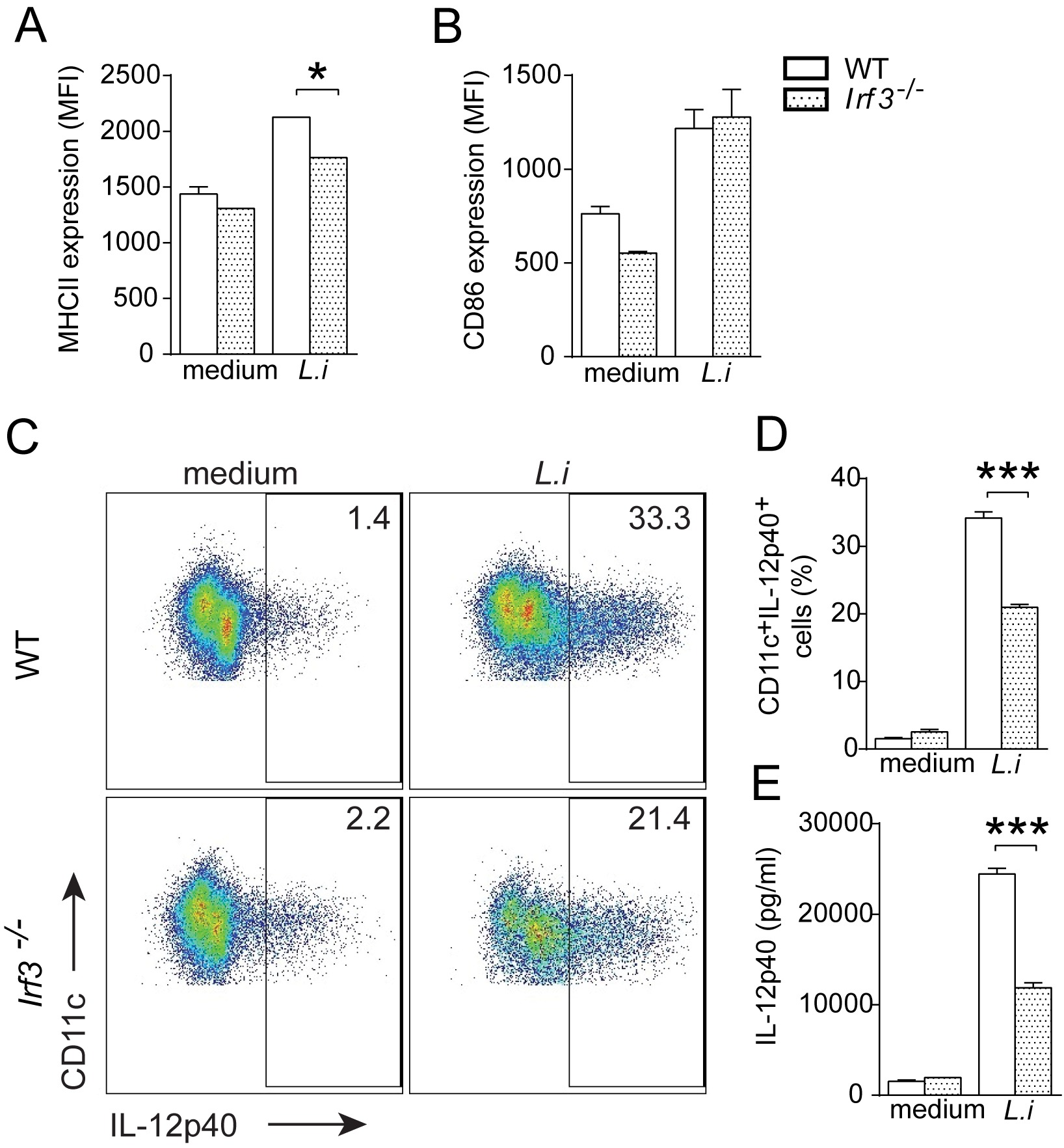

Supplement: S5 Fig — WT and Irf3-/- BMDCs were infected with L. infantum (5 parasites:1 cell) or not infected (medium) for 24 h. Graph bars of the MFI of MHCII (A) and CD86 (B) are shown. (C) Representative dot plots showing the production of IL-12p40 by CD11c+ cells. (D) Graph bars showing the percentage of CD11c+ IL-12p40+ cells. (E) The IL-12p40 levels in culture supernatants were measured by ELISA. The data are expressed as the means ± SEMs (BMDCs were assessed in quadruplicate). The statistical significance was calculated by one-way ANOVA with the Bonferroni post hoc test (*p < 0.05 and ***p < 0.001). (TIF) [file ppat.1008435.s005.tif]
