# Supplementary material for: TLR4 abrogates the Th1 immune response through IRF1 and IFN-β to prevent immunopathology during L. infantum infection
Source: PLoS Pathog. 2020 Mar 25;16(3):e1008435. doi: 10.1371/journal.ppat.1008435 (PMC7135367; doi:10.1371/journal.ppat.1008435)
Supplement: S1 Protocol — (DOCX) [file ppat.1008435.s007.docx]

**SUPPORTING PROTOCOL**

*Flow cytometry assay*

For leukocyte identification, the inflammatory cells isolated from the spleen and liver were first gated based on their characteristic size (FSC) and granularity (SSC), and the T lymphocytes (CD3^+^CD4^+^ cells), B lymphocytes (CD19^+^ cells), macrophages (CD11b^+^F4/80^+^ cells) and dendritic cells (DCs, CD11b^+^CD11c^high^) were identified individually. The total leukocyte counts were determined by measuring the relative expression of the leukocyte subpopulations stained with a specific antibody within a set of 300,000 acquired events as a proportion of the leukocyte number obtained in a Neubauer chamber. Antibodies against the following mouse proteins as well as their respective isotype controls were obtained from BioLegend: CD3 (145-2C11), CD4 (RM4-5), CD19 (1D3), IFN-γ (XMG1.2), CD11b (M1/70), CD11c (N418), F4/80 (BM8), MHC class II (M5/114.15.2), CD40 (3.23), CD86 (GL-1), IL-12p40 (C15.6), T-bet (4B10), TLR4 (SA15-21), PD-L1 (10F.9G2) and CTLA-4 (UC10-4B9).

*Transaminase measurement*

For measurement of the AST and ALT levels in serum, blood from naïve and infected mice was centrifuged at 8000 rpm for 10 min to separate the serum. The kinetic of the transaminase levels was assessed based on the activation of pyridoxal phosphate. The colorimetric reaction was measured at 340 nm using a microplate reader (EMAX, Molecular Devices Corporation).

*Quantitative real-time PCR*

Total RNA was isolated from DC cultures, the spleen or the liver using the SV Total RNA Isolation System Kit (Promega, Madison, WI, USA), and complimentary DNA (cDNA) was generated using Transcriptase Reverse SuperScript III (InvivoGen). SYBR Green Mix-based real-time qPCR assays were performed using the StepOnePlus Real-Time PCR System (Applied Biosystems). The gene expression levels in the spleen and in the liver and DC cultures were normalized to the glyceraldehyde-3-phosphate dehydrogenase (GAPDH) expression level and the Rpl13 ribosomal protein L13 (RPL13a) expression level, respectively.

To analyze the transcriptional pattern of genes related to TLR signaling, we used an RT^2^ Profiler PCR array from Qiagen. The PCR plate has predefined genes and thus allows simultaneous analyses of pathway-focused gene expression. The PCR arrays were performed using samples from BMDCs stimulated for 4 h in quadruplicate. SYBR Green Master Mix-based reactions were performed in 96-well plates: 84 wells contained the target genes, six wells contained housekeeping genes, three wells contained reverse transcription controls, and three wells contained positive controls. The data were analyzed using the clustered distance (hclust, gplot from R package) and 2-fold changes (positive or negative) in the expression values of the transcripts.

| Table 1. Primer sequences used in the study | | |
| --- | --- | --- |
| Gene | Forward (5’-3’) | Reverse (5’-3’) |
| Mu GAPDH | TGCAGTGGCAAAGTGGAGAT | CGTGAGTGGAGTCATACTGGAA |
| Mu RPL13a | GGAGGAGAAACGGAAGGAAAAG | TTTCCGTAACCTCAAGATCTGCTT |
| Mu TLR4 | CCAAGCCTTTCAGGGAATTAAG | GCCAGGTTTTGAAGGCAAGT |
| Mu IRF1 | CGGCTGGGACATCAACAAG | TTGGGATCTGGCTCTTTTTTCTC |
| Mu IFN-β | CATCAACTATAAGCAGCACCA | TTGGGATCTGGCTCTTTTTTCTC |
| *Mu, sequence of murine primer* | | |

*SDS-PAGE and immunoblot analysis*

After 8 h of stimulation, total BMDC lysates were prepared and equalized based on the total protein concentration as previously described ([1](#_ENREF_1)). The protein homogenates were mixed with sample buffer [4% SDS, 160 mM Tris-HCl (pH 6.8), 20% glycerol, 100 mM DTT and 0.005% bromophenol blue] and boiled. The proteins were resolved by SDS-PAGE under reducing conditions and electrotransferred to nitrocellulose membranes (Millipore, Bedford, MA, USA). The blots were probed with α-IRF-1 (1:1000, D5E4, #8478, Cell Signaling Technology) and α-β-actin (1:1000, C4; sc-47778, Santa Cruz Biotechnology) primary antibodies and appropriate HRP-conjugated secondary antibodies. The proteins in the blots were visualized by enhanced chemiluminescence using ECL (GE Healthcare).

*Immunofluorescence microscopy*

BMDCs were cultured on glass coverslips. After 24 h of stimulation, the cells were fixed for 15 min at room temperature with 4% (w/v) paraformaldehyde (PFA) in PBS. The PFA-fixed cells were permeabilized with 0.01% (w/v) saponin in blocking solution [0.2% (w/v) pork skin gelatin in PBS] for 15 min at 37°C and labeled with α-IRF1 antibody (1:50, D5E4, #8478, Cell Signaling Technology) and then with α-IgG antibody (1:1000, Life Technologies). The cells were imaged using a Zeiss confocal laser-scanning microscope (LSM) 780 (Zeiss, Jena, Germany). Postacquisition image processing and colocalization analysis were performed as previously described ([1](#_ENREF_1)).

*Patients*

Peripheral blood specimens were collected from nine (male, N=5 males; female, N=4) healthy individuals (age in years [average ± SD] = 24 ± 5), nine (male, N=6; female, N=3) asymptomatic individuals (age in years [average ± SD] = 20 ± 16) and 12 (male, N=6; female, N=6) patients with VL (age in years [average ± SD] = 16 ± 13; characterized by the presence of fever, weight loss, hepatosplenomegaly, and low leukocyte and platelet counts) using Vacutainer tubes (BD Biosciences, San Diego, CA, USA). Endemic asymptomatic subjects were defined as healthy individuals who presented no alterations in hematological tests and no clinical signs of VL but positive reactions to leishmanial antigens (Montenegro Skin test and rK39 serological test). Thus, these individuals were considered to have been infected with *L. infantum* but never experienced the outcome of the infection (VL disease). Each VL diagnosis was confirmed by observation of *Leishmania* parasites in the bone marrow or a positive result from an rK39 serological test (Kalazar Detect Rapid Test, InBios, Seattle, WA, USA). All patients were negative for hepatitis B and C viruses and HIV, and none of the patients had diabetes. Total RNA samples from white blood cells were purified using the PAXgene Blood RNA Kit (PreAnalytix, BD) and the GLOBINclear-Human Kit (Applied Biosystems/Ambion, Singapore). The samples were quantified using a Qubit 3.0 fluorometer (Thermo Fisher Scientific, Waltham, MA, USA), and the RNA integrity was assessed with the lab-on-a-chip Agilent 2100 Bioanalyzer.

VL patients presented low white blood cell counts, including lymphocytes (Table 2). Important normalization steps during the RNA-seq experiment were performed to guarantee a homogeneous composition among the samples. First, cDNA libraries were constructed for all the samples using the same amount of RNA (300 μg of RNA depleted from ribosomal RNA and mRNA of globins). Second, the cDNAs libraries were quantified by qPCR before sequencing to ensure that the same amount of cDNA from the various sample would be sequenced. Third, the statistical methods for the assessment of differential expression employed in this study were performed using the edgeR package: the samples were normalized by adjusting the sequencing depth based on the different library sizes (due to a technical factor during sequencing), and the RNA composition was adjusted by identifying a set of scaling factors for the library sizes that would minimize the log-fold changes between the samples for most genes, e.g., a measure of the RNA output on a per-cell basis. Ultimately, all these normalizations ensure that the differences in expression levels observed between the groups are due to the biological properties of the samples, i.e., disease state, rather than to the analysis of different numbers of cells (S1 Table). Furthermore, the DEG data showed that more than half of the differentially expressed transcripts were upregulated (2325), and approximately 13% of these upregulated transcripts showed highly differentially expression with a fold increase greater than 4 (log_2_FC > 2). To confirm the reliability of the normalization procedures used to minimize the effect of the cell number, we plotted the expression levels of the most suitable endogenous reference genes for gene expression studies of human peripheral blood. The expression of the selected endogenous reference genes for peripheral blood, namely, DECR1, TRAP1 and FPGS ([2](#_ENREF_2)), presented similar average expression values in all the groups, and no significant difference in their expression was found between the groups; these findings confirm that the groups being compared exhibited transcript contents that were quantitatively equivalent.

| Table 2. Clinical characteristics of the studied subjects. | | | |
| --- | --- | --- | --- |
|  | **Control (n = 9)** | **Asymptomatic (n = 9)** | **VL patients (n = 12)** |
| Gender (M/F) | 5/4 | 6/3 | 6/6 |
| Mean age (min/max) | 23(11/29) | 19.87(7/42) | 16.36(1/44) |
| **Clinical data**  **(mean ± SD)** |  |  |  |
| Spleen enlargement  (cm) | 0 ± 0 | - | 16.5 ± 6.89 |
| Liver enlargement  (cm) | 0 ± 0 | - | 6.58 ± 4.86 |
| **Hematological data**  **(mean ± SD)** |  |  |  |
| Leukocytes (/mm3) | - | - | 2751.81 ± 1172.15 |
| Neutrophils (/mm3) | - | - | 991.81 ± 684.75 |
| Eosinophils (/mm3) | - | - | 6 ± 9.68 |
| Lymphocytes (/mm3) | - | - | 1467.88 ± 857.30 |
| Monocytes (/mm3) | - | - | 394.44 ± 221.83 |
| Hemoglobin (g/dL) | - | - | 7.84 ± 0.90 |
| Platelet (/mm3) | - | - | 120990.90 ± 51622.87 |

*RNA sequencing*

RNA-seq raw data were generated using the Illumina HiSeq 2500 platform (Illumina, San Diego, CA, USA) at the Genomics Center of the Laboratory of Animal Biotechnology (Piracicaba, Brazil) according to previously described procedures ([3](#_ENREF_3)). Briefly, libraries were prepared using a TruSeq Stranded RNA Sample Preparation kit with Poly(A)+ selection, quantified by qPCR, and sequenced using a HiSeq SBS V4 kit for paired-end reads. The resulting Fastq files were checked for quality control using FastQC ([4](#_ENREF_4)) and trimmed using Trimmomatic ([5](#_ENREF_5)). STAR aligner ([6](#_ENREF_6)) was used to map the reads to the human genome reference assembly GRCh38 (Ensembl release 84, provided by the GENCODE project) and to quantify the read counts. Differential expression analyses were performed with the edgeR package ([7](#_ENREF_7), [8](#_ENREF_8)) using a threshold false discovery rate (FDR) of < 0.05.

Gene set enrichment analyses were performed using the edgeR built-in roast function and gene signatures from ImmuneSigDB ([9](#_ENREF_9)), namely, GSE16755 (GSE16755_CTRL_VS_IFNA_TREATED_MAC_UP) for human macrophage gene signatures associated with IFN-I stimulation ([10](#_ENREF_10)) and GSE9988 (GSE9988_LOW_LPS_VS_VEHICLE_TREATED_MONOCYTE_DN) for human monocyte gene signatures associated with TLR4 activation by LPS stimulation ([11](#_ENREF_11)). The RNA-seq data are available in the ArrayExpress database (http://www.ebi.ac.uk/arrayexpress under accession number E-MTAB). A heatmap was prepared by hierarchical clustering using the Euclidean distance for the metric calculations with MultiExperiment Viewer (MeV) software ([12](#_ENREF_12)). The color scale represents the z-score calculated from the log2 transformation of the counts per million reads obtained from the normalized RNA sequencing libraries, and the averages from each group were plotted.

**REFERENCES**

1. Amorim NA, da Silva EM, de Castro RO, da Silva-Januário ME, Mendonça LM, Bonifacino JS, et al. Interaction of HIV-1 Nef protein with the host protein Alix promotes lysosomal targeting of CD4 receptor. Journal of Biological Chemistry. 2014;289(40):27744-56.

2. Ohl F, Jung M, Radonić A, Sachs M, Loening SA, Jung K. Identification and validation of suitable endogenous reference genes for gene expression studies of human bladder cancer. The Journal of urology. 2006;175(5):1915-20.

3. Nascimento MS, Ferreira MD, Quirino GF, Maruyama SR, Krishnaswamy JK, Liu D, et al. NOD2-RIP2–Mediated Signaling Helps Shape Adaptive Immunity in Visceral Leishmaniasis. The Journal of infectious diseases. 2016;214(11):1647-57.

4. Lin X, Sun D, Rodriguez B, Zhao Q, Sun H, Zhang Y, et al. BSeQC: quality control of bisulfite sequencing experiments. Bioinformatics. 2013;29(24):3227-9.

5. Bolger AM, Lohse M, Usadel B. Trimmomatic: a flexible trimmer for Illumina sequence data. Bioinformatics. 2014;30(15):2114-20.

6. Dobin A, Davis CA, Schlesinger F, Drenkow J, Zaleski C, Jha S, et al. STAR: ultrafast universal RNA-seq aligner. Bioinformatics. 2013;29(1):15-21.

7. Robinson MD, McCarthy DJ, Smyth GK. edgeR: a Bioconductor package for differential expression analysis of digital gene expression data. Bioinformatics. 2010;26(1):139-40.

8. McCarthy DJ, Chen Y, Smyth GK. Differential expression analysis of multifactor RNA-Seq experiments with respect to biological variation. Nucleic acids research. 2012;40(10):4288-97.

9. Godec J, Tan Y, Liberzon A, Tamayo P, Bhattacharya S, Butte AJ, et al. Compendium of immune signatures identifies conserved and species-specific biology in response to inflammation. Immunity. 2016;44(1):194-206.

10. Greenwell-Wild T, Vázquez N, Jin W, Rangel Z, Munson PJ, Wahl SM. Interleukin-27 inhibition of HIV-1 involves an intermediate induction of type I interferon. Blood. 2009;114(9):1864-74.

11. Dower K, Ellis DK, Saraf K, Jelinsky SA, Lin L-L. Innate immune responses to TREM-1 activation: overlap, divergence, and positive and negative cross-talk with bacterial lipopolysaccharide. The Journal of Immunology. 2008;180(5):3520-34.

12. Saeed A, Sharov V, White J, Li J, Liang W, Bhagabati N, et al. TM4: a free, open-source system for microarray data management and analysis. Biotechniques. 2003;34(2):374.
